# RNA degradomes reveal substrates and importance for dark and nitrogen stress responses of Arabidopsis XRN4

**DOI:** 10.1093/nar/gkz712

**Published:** 2019-08-20

**Authors:** Vinay K Nagarajan, Patrick M Kukulich, Bryan von Hagel, Pamela J Green

**Affiliations:** Delaware Biotechnology Institute and Department of Plant and Soil Sciences, University of Delaware, Newark, DE 19711, USA

## Abstract

XRN4, the plant cytoplasmic homolog of yeast and metazoan XRN1, catalyzes exoribonucleolytic degradation of uncapped mRNAs from the 5′ end. Most studies of cytoplasmic XRN substrates have focused on polyadenylated transcripts, although many substrates are likely first deadenylated. Here, we report the global investigation of XRN4 substrates in both polyadenylated and nonpolyadenylated RNA to better understand the impact of the enzyme in Arabidopsis. RNA degradome analysis demonstrated that *xrn4* mutants overaccumulate many more decapped deadenylated intermediates than those that are polyadenylated. Among these XRN4 substrates that have 5′ ends precisely at cap sites, those associated with photosynthesis, nitrogen responses and auxin responses were enriched. Moreover, *xrn4* was found to be defective in the dark stress response and lateral root growth during N resupply, demonstrating that XRN4 is required during both processes. XRN4 also contributes to nonsense-mediated decay (NMD) and *xrn4* accumulates 3′ fragments of select NMD targets, despite the lack of the metazoan endoribonuclease SMG6 in plants. Beyond demonstrating that XRN4 is a major player in multiple decay pathways, this study identified intriguing molecular impacts of the enzyme, including those that led to new insights about mRNA decay and discovery of functional contributions at the whole-plant level.

## INTRODUCTION

Cytoplasmic mRNA decay is an important post-transcriptional step for controlling gene expression and eliminating undesirable or aberrant RNA. It is a complex process involving multiple pathways (1–3). A key first step in the process for many eukaryotic mRNAs is deadenylation down to an oligoA tail of about 15 nucleotides or less (1,3). Deadenylated RNA can either be degraded in the 3′ to 5′ direction by the multimeric exosome complex or 5′ to 3′ after decapping (2). RNA can also be degraded in the 3′ to 5′ direction by exoribonuclease SOV (DIS3L2) (4–6). The decapping complex (DCP1/DCP2/VCS) removes the cap structure, producing a 5′ monophosphorylated RNA, the preferred substrate of the major cytoplasmic 5′ to 3′exoribonuclease XRN1 or, in plants, XRN4 (3,7). Alternative pathways exist in which transcripts bypass deadenylation and undergo decapping followed by decay by XRN1 (e.g. during nonsense-mediated decay, NMD) or are cleaved internally (e.g. during small RNA-directed argonaute (AGO) cleavage) and are degraded exonucleolytically from the cleaved ends (2,8–13).

XRN4, discovered in Arabidopsis, is the plant functional homolog of XRN1. Although it is an ortholog of the nuclear XRN2/RAT1 of metazoans and yeast, XRN4 has a deletion in the nuclear localization sequence and resides in the cytoplasm (12,14). Accordingly, XRN4 has 5′ to 3′ exoribonuclease activity when expressed in yeast and in contrast to the Arabidopsis nuclear enzymes, XRN2 and XRN3, fails to complement an *xrn2/rat1* mutant (14–16). It was the functional characterization of Arabidopsis XRN4 that led to the first report of an XRN enzyme degrading downstream intermediates from small RNA-guided cleavage, a function common to XRN1 (12,17).

In contrast to animals, plant miRNAs typically guide cleavage of their target by an AGO protein that generates a 5′ monophosphorylated downstream fragment that can be validated by sequencing. To do this on a global scale in wild-type (WT) and *xrn4* mutant Arabidopsis plants, parallel analysis of RNA ends (PARE) was developed (18,19). Although most previously validated miRNA targets could be detected by PARE in WT, *xrn4* increased the sensitivity of detection for some that were otherwise amidst the background (19,20).

One prominent process of mRNA decay involving at least XRN1 is nonsense-mediated decay (NMD) (21–26). Aberrant mRNAs with premature termination codons (PTCs) are the most well-known targets of NMD. These transcripts are usually subject to 5′ to 3′ decay following decapping in fungi or following endoribonucleolytic cleavage by SMG6 (or decapping) in metazoans (27–30) prior to XRN1-mediated decay (9). Although SMG6 is lacking in both plants and fungi, the universal NMD factor UPF1 is essential for NMD in eukaryotes in general. NMD can also target normal transcripts with other features such as introns downstream of stop codons, long 3′ UTRs, or upstream ORFs (uORFs, some of which are conserved peptide uORFs and designated CPuORFs) (21,26,31). These features are often found in transcripts elevated in Arabidopsis *upf1* mutants (31,32). Recently, it was proposed that XRN4 does not play a major role in plant NMD because expression of PTC-containing reporter transcripts appeared unchanged in an *XRN4*-silenced line of *Nicotiana benthamiana* (33). However, given that all NMD targets do not require all the same NMD factors for their degradation (34–36) and some plants have different complements of NMD factors (26,33,37–39), the study of endogenous NMD targets in *xrn4* is warranted.

**Figure 1.**
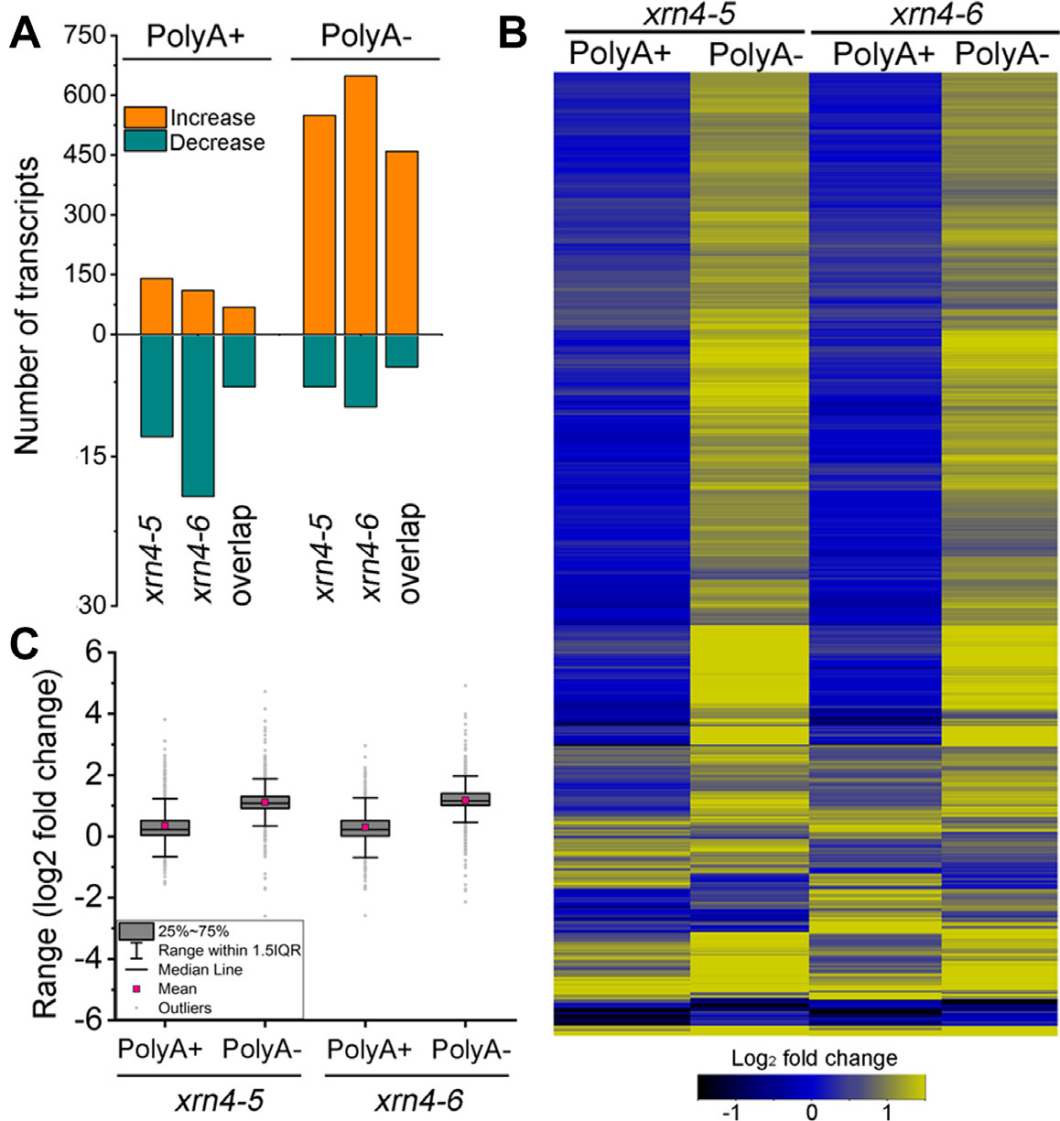
Elevation of many nonpolyadenylated transcripts in *xrn4* seedlings. (**A**) Histograms show the number of protein-coding transcripts that are either increased or decreased (≥2 fold i.e. log_2_≥ ±1); FDR-adjusted *P*< 0.05) in two *xrn4* mutants relative to the WT (Col-0) in polyA+ and polyA- RNA-seq. Overlap, number of differentially accumulating transcripts that are in common between the mutants. (**B**) The heat map illustrates the fold changes in the *xrn4* mutants of differentially accumulating transcripts (Supplementary Dataset S1). (**C**) Box plots show the range of fold changes in *xrn4* mutants for differentially accumulating transcripts described in panel (B).

In contrast to NMD, which is often initiated without deadenylation, the first step in current models for the turnover of many mRNAs degraded by XRN1 or XRN4 is deadenylation (see Figure 1 in 2,3,40–42). Yet transcriptomic approaches, such as microarray, tiling array and RNA-seq, have nearly always relied on polyadenylated RNA and occasionally total RNA to investigate cytoplasmic XRNs. The situation is similar for RNA degradome approaches including PARE, GMUCT (Genome-wide mapping of uncapped and cleaved transcripts), 5′Seq etc., except that the use of total RNA is much more limited and no study has included separate polyadenylated and nonpolyadenylated mRNA fractions (12,19,43–51). In particular, an in-depth analysis of deadenylated RNA has the potential to reveal novel substrates of XRN4. In either RNA fraction, XRN4 substrates should overaccumulate in *xrn4* mutants after they are decapped.

At the organismal level, loss of XRN4 has minimal effects under normal physiological conditions. Changing the growth conditions has revealed deficiencies, for example, with respect to hormones. Loss-of-function mutants of *XRN4* are also referred to as *ein5* or *ain1*, in addition to *xrn4*, because they are insensitive to ethylene (12,16,52,53). The phenotype is likely due to elevated levels of *EIN3 BINDING F-BOX PROTEIN1* (*EBF1)* and *EBF2*, possibly as a result of reduced 3′UTR-dependent translational repression of these mRNAs (54,55). It has been reported that *xrn4* mutants also display altered sensitivity to auxin and abscisic acid (ABA) treatments (56,57). XRN4 is also critical for the response to heat stress and the accelerated decay of a number of RNAs (49,58).

The hypothesis underlying this work was that the identification of XRN4 substrates on a global scale would enable the discovery of new biological impacts of the enzyme and lead to new post-transcriptional mechanistic insights. Here, we report on the substrates and functional importance of XRN4 identified by analyzing the RNA transcriptome and degradome of Arabidopsis seedlings using polyadenylated and nonpolyadenylated RNA. Transcripts with shortened polyA tails (deadenylated) represented the majority of XRN4 substrates. The use of PARE and a variation called Cap-PARE (C-PARE) facilitated the identification of many deadenylated and polyadenylated substrates of XRN4 that were decapped, having 5′ ends precisely corresponding to cap sites. Prominent among these were transcripts known to function in photosynthesis and during responses to nitrogen (N), hormones and environmental stresses. Further, we show the loss of XRN4 results in increased sensitivity of Arabidopsis to prolonged darkness. XRN4 was also found to be required for normal lateral root responses during N resupply. The degradomes from polyadenylated and deadenylated RNA provided other insights, including clear evidence for involvement of XRN4 in NMD. Transcripts with CPuORFs and those elevated in *upf1* and *upf3* were found among XRN4 decapped substrates. Further, *xrn4* overaccumulates polyadenylated 3′ fragments of some NMD targets reminiscent of SMG6 cleavage products, an observation not previously reported in plants.

## MATERIALS AND METHODS

### Plant materials and growth conditions

Arabidopsis (*Arabidopsis thaliana*) ecotype Col-0 (WT), *XRN4* T-DNA insertion lines (*xrn4–5* (59) and *SALK_014209* (60)), *xrn2–1xrn3–3* (60) and *upf1–1* (61) in the Col-0 background were used. In several studies (58,62–64) including ours, SALK_014209 is referred to as *xrn4–6* but is also called *xrn4–3* in other studies (53,65,66). Seeds were surface-sterilized with 70% (v/v) ethanol followed by 50% (v/v) commercial bleach. After 2 days of stratification at 4°C, surface sterilized seeds were plated on germination medium containing, 1× Murashige and Skoog (MS) salts, 1× B5 Vitamin mix, 1.0% (w/v) sucrose, 0.5% (w/v) 2-(N-morpholino)ethanesulfonic acid (MES) and 1% (w/v) agar, adjusted to pH 5.7 (46). Seeds were germinated and grown for 14 d under long-day conditions of 16-h light/8-h dark at 21°C. For the dark experiments, 14 d plants were wrapped in aluminum foil and placed in a closed box for duration indicated, while control plants were grown in normal long-day conditions. For dark-induced senescence assays, fully expanded rosette leaves from 4-week-old soil-grown plants were detached and incubated in Petri plates with filter paper pre-wetted with 3 mM MES-KOH adjusted to pH 5.7. Plates were left at 21°C for 4 days under photoperiod control conditions or wrapped in aluminum foil and left in darkness. For N starvation and resupply, all plants were grown vertically on 1.2% (w/v) agar-solidified media. Uniformly grown seedlings (∼2 cm of primary root length) after an initial 7 d growth on germination medium were transferred to modified MS as described previously (67), i.e. N-replete medium with N in the form of 2.0 mM NH_4_NO_3_ and 1.9 mM KNO_3_ and N-starvation medium (substituting N with equimolar KCl) for 7 d. Seedlings were then transferred back to N-replete medium as N-resupply for 7 d. After resupply, roots were carefully spread to reveal architectural details and scanned at 600 dpi. The ImageJ program (imagej.nih.gov/ij/) was used to analyze lateral root number and primary root length. Soil-grown plants were used for harvesting mixed stage inflorescence (46) after 5 weeks of growth in a chamber under normal long-day conditions as mentioned above. For RNA half-life experiments, 2-week-old WT and *xrn4–5* seedlings were transferred to a liquid germination medium as described above. After a 30-min incubation, cordycepin (3-deoxyadenosine, Sigma-Aldrich) was added to the medium to a final concentration of 0.6 mM and vacuum infiltrated for 45 s (at time 0). Tissue samples were harvested at regular intervals thereafter and frozen in liquid nitrogen.

### Library construction and analysis

Construction, sequencing summary statistics and analysis of RNA-seq and PARE libraries are described in Supplementary Experimental Procedures.

### RNA analysis

#### Northern blots

Total RNA (10 μg) and/or fractionated RNA (polyA+ and/or polyA-) were resolved on 1.2% formaldehyde-agarose denaturing gels. Blots were transferred to Hybond N+ membrane and hybridizations were carried out in a sodium phosphate buffer (68) overnight at 65°C with random-primed ^32^P-dCTP labeled DNA probes. All membranes were washed with 2× SSC, 0.1% SDS for 10 min at 65°C followed by one or two washes of 0.2× SSC, 0.1× SDS for 10 min at 65°C. The Typhoon system (GE Health Sciences) was used for analyzing signal intensities. Membranes were stripped at least twice in boiling 0.1% SDS for 20 min between hybridizations. All northern blot results presented are representative of two independent biological experiments.

#### Quantitative RT-PCR

Total (1 μg) or polyA+ RNA (100 ng) was reverse-transcribed with random decamer primer using SuperScript II. qRT-PCR was performed on BioRad CFX96 Touch Real-Time PCR using Premix Ex Taq SYBR Green (Clontech Inc.). Relative levels were computed by the 2^−ΔΔ*C*t^ method of quantification (69) and normalized to *UBC* (AT5G25760) (70). All qRT-PCR experiments were performed with three technical replicates from two or more biological replicates as per the corresponding figure legends. Statistical differences between means were determined using nonparametric Mann–Whitney *U* test. Splinted-ligation assays are described in the Supplementary Experimental Procedures.

#### RNA half-life

Half-lives were determined essentially as described by (71). The rate of RNA decay (*k*_decay_) was calculated by regression analysis across multiple time points (0, 15, 30 and 60 min) and half-lives (*t*_1/2_) were calculated by the equation *t*_1/2_ = 0.693/*k*_decay_.

### Chlorophyll estimation

Total chlorophyll from leaves or whole seedlings were extracted in the dark using 100% methanol for 16 h with gentle shaking at 4°C. The extracts were then diluted (1:1) in methanol and absorbance was measured at OD_652_ and OD_665_, where OD is optical density. Total chlorophylls (Chls) were estimated as described (72) with the following equation: Total Chls *a* + *b* (μg /ml) = (22.12 OD_652_ + 2.71 OD_665_), normalized to g fresh-weight.

## RESULTS

### Many deadenylated mRNAs are elevated in mutants lacking XRN4

To better understand the impact of XRN4 in Arabidopsis seedlings, endogenous substrates of the enzyme were investigated on a global scale. We postulated that XRN4 substrates may be prominent in the deadenylated component of the RNA degradome, a situation that would necessitate new RNA degradome methodology. Therefore, at the first step a simpler RNA-seq experiment was used to evaluate the deadenylated transcriptome. Strand-specific libraries were made from separate polyA+ (polyadenylated) and polyA- (nonpolyadenylated) RNA fractionated from the total RNA of WT (Col-0) and two well-studied T-DNA insertion mutants of *XRN4, xrn4–5* and *xrn4*–6. (Supplementary Experimental Procedures). This approach would not report on XRN4 substrates directly. However, the distribution of overaccumulating transcripts in *xrn4* mutants would indicate if polyA+ and polyA- types of RNA degradome libraries should be valuable for doing so.

The RNA-seq data were analyzed using the Tuxedo suite (73,74). Differentially accumulating protein-coding transcripts in each mutant relative to the WT were identified from each experiment (polyA+ or polyA-). In the polyA+ libraries, 140 and 110 transcripts were elevated compared to the WT in *xrn4–5* and *xrn4–6*, respectively (Figure 1A and Supplementary Table S1). The low numbers of polyA+ transcriptomic changes (ranging from 22 to 156) in *xrn4* mutants are consistent with previous reports (12,16,44–46). In contrast, in the polyA- transcriptome, 549 and 648 transcripts showed enhanced accumulation relative to WT in *xrn4–5* and *xrn4–6*, respectively (Figure 1B). For all the differentially accumulating transcripts, the average fold change in abundance between *xrn4* and WT was also greater in the polyA- (median ∼ 2.1 fold) than polyA+ (median ∼ 1.2-fold) libraries (Figure 1B and C). Of the transcripts elevated in both *xrn4* mutants by ≥2 fold, 68 were from polyA+ libraries and 459 were from polyA- (Supplementary Table S1). These results indicate that a large majority of the protein-coding transcripts that overaccumulate in *xrn4* are in the polyA- fraction.

To validate several of these transcripts identified by RNA-seq as overaccumulating in *xrn4*, northern blots of total RNA were compared alongside fractionated (polyA+ and polyA-) RNA in biological replicates different from those used for the RNA-seq (Supplementary Figure S1). The controls, *eIF-4A* mRNA and nonpolyadenylated *AT7SL* ncRNA, fractionated specifically to polyA+ and polyA-, respectively, demonstrating the efficacy of the fractionation (Supplementary Figure S1 and S2). Transcripts that showed increases in abundance in *xrn4* of ≥2 fold in polyA- (*IAA2, SAUR50, AT5G20885* and *CAB2*) or in both polyA+ and polyA- (*RAP2.4* and *AT4G32020*) and those unchanged (*AT7SL* and *eIF-4A*) were examined. All were found to increase in *xrn4*, or remain unchanged (for *AT7SL* and *eIF-4A*), in the expected fractions. Overall, the results from RNA-seq were in close agreement with those observed in the northern blot experiments.

The transcripts in polyA- fraction in *xrn4* appeared full length in the northern blots and this was consistent with circular RT-PCR experiments (cRT-PCR) designed also to examine their polyA tail lengths. In these experiments, polyA- RNA was circularized by ligation and the ligated junctions for several genes were reverse transcribed, amplified and sequenced. The data indicated that the transcripts were full length; sequences expected for the 3′ ends were followed by oligoA sequences that were adjacent to sequences at, or close to, the annotated 5′ ends (Supplementary Table S2). The average median polyA length was found to be 12 nt, which is consistent with the oligoA that remains after deadenylation in previous studies (42,66,75–78). Therefore, deadenylated RNAs are included in the polyA- transcriptome and are elevated in the *xrn4* mutants (Figure 1; Supplementary Figure S1; Supplementary Dataset S1). cRT-PCR products were also examined in both WT and *xrn4–5* for a subset of genes and polyA tail lengths were comparable. Classical 5′ RACE was also used to map the 5′ ends of the polyA- transcripts and the data were consistent with the cRT-PCR. The top 5′ end in the cRT-PCR was the same as that either in the 5′ RACE or in the top two (Supplementary Table S2). These data indicate that the RNAs in Supplementary Figure S1 are full length and the corresponding polyA+ and polyA- RNA fractions are good starting material to examine decapped and other RNA decay intermediates on a global scale.

### PARE captures polyA+, polyA- and decapped RNA decay intermediates of known miRNA targets

To explore RNA decay intermediates more directly, we employed PARE, a well-established deep-sequencing approach, which detects partially degraded mRNAs that have a monophosphate at the 5′ end (18,19). Originally developed to sequence cleaved miRNA targets in polyA+ RNA, the PARE approach was modified to also capture separately deadenylated RNA (Supplementary Figure S2A). This was accomplished by separating the polyA- RNA and A-tailing before the RNA was processed as for polyA+ PARE (Supplementary Experimental Procedures). As mentioned earlier, XRN4 is known to degrade the 3′ fragment resulting from miRNA-guided cleavage of select target RNAs (12,19,45,46). Thus, the PARE libraries made from polyA+ and polyA- RNA from two *xrn4* mutants (*xrn4–5* and *xrn4–6*) and two replicates of the WT that were grown alongside provide the means to extend knowledge about this set of XRN4 substrates and to identify others.

**Figure 2.**
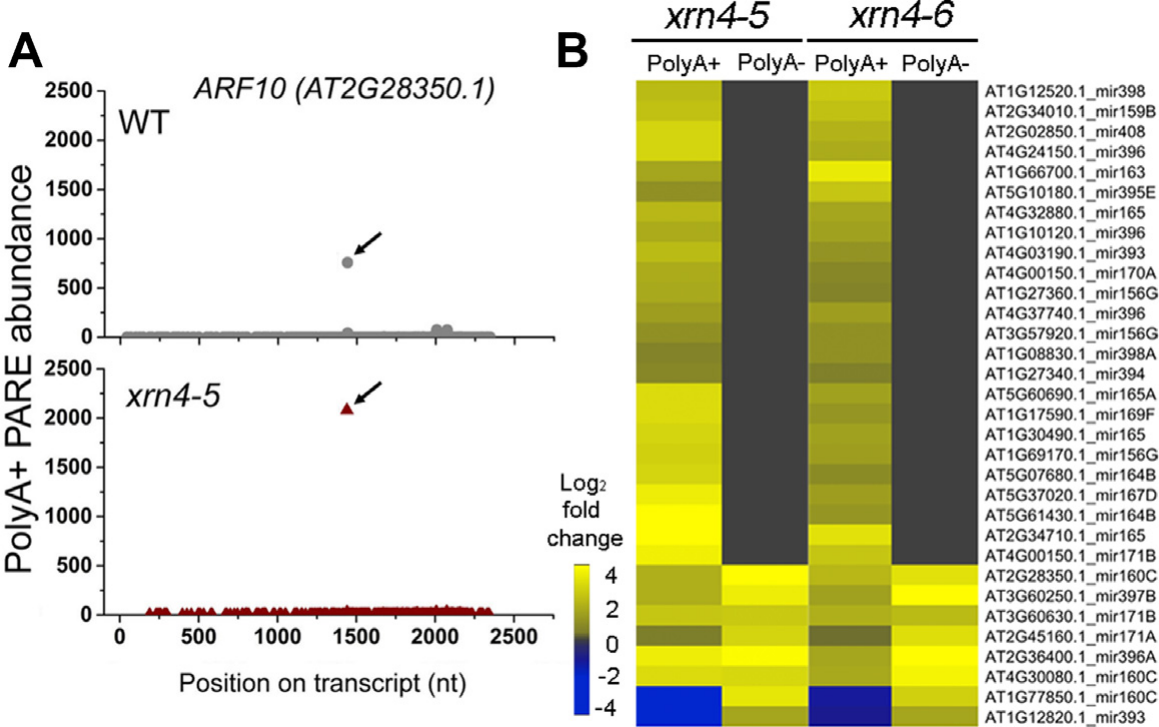
Most 3′ fragments of cleaved miRNA targets overaccumulate in *xrn4* as polyadenylated RNA. (**A**) Decay plots (D-plots) of a validated miRNA target transcript *ARF10* that overaccumulates 3′ fragment of miR160-guided cleavage in *xrn4–5*. Black arrow, miRNA-guided cleavage site. (**B**) The heat map of miRNA targets shows fold changes of 3′ fragment abundance in *xrn4* in polyA+ and polyA- PARE (Supplementary Table S3). Abundance at cleavage site (±1 nt) in both *xrn4* mutants ≥ 10 TP20M and ≥1.5-fold change.

We first examined whether the known accumulation of the miRNA-guided cleavage intermediates of *ARF10* mRNA in *xrn4* mutants reported previously could be recapitulated with PARE*. xrn4* had been shown to overaccumulate the 3′ fragment of cleaved *ARF10* mRNA (12,46). As shown in the polyA+ decay plots (D-plots) (Figure 2A), the most abundant PARE sequence from the *ARF10* mRNA matches the 5′ end of the miRNA cleavage fragment. This PARE sequence is markedly elevated in *xrn4–5* (and in *xrn4–6*, Supplementary Figure S4A), as seen previously for the corresponding cleavage fragment in northern blots (12,46). The data show that PARE reproduces the characteristic accumulation pattern of the *ARF10* fragment in *xrn4* seedlings.

The analysis was extended to include 138 miRNA targets whose cleavage had been experimentally validated (20). PARE sequences at cleavage sites of miRNA targets that overaccumulated in both *xrn4*–*5* and *xrn4–6* by ≥1.5-fold compared to WT were identified. Twenty-nine of these overaccumulated in polyA+ RNA, whereas only seven did in polyA- RNA, including four which overlapped the polyA+ set (Figure 2B and Supplementary Table S3). For the remaining miRNA targets, the abundance of cleavage intermediates at the predicted site was either comparable between the WT and *xrn4* mutants or no sequences were detected at the cleavage site. *ARF6* mRNA, targeted by miR167, is an example of the larger group of miRNA targets that overaccumulate the 3′ intermediate beginning at the miRNA cleavage site in *xrn4* polyA+ RNA (Figure 2B) but not in polyA- RNA (Supplementary Figure S4B). In the case of the *GRF3*, the PARE sequence at the miR396 cleavage site is elevated in *xrn4* in both polyA+ and polyA- RNA (Figure 2B and Supplementary Figure S4C). In addition, a decapped deadenylated intermediate of *GRF3* mRNA (i.e. with its 5′ end precisely corresponding to the cap site determined with C-PARE as described below) also preferentially accumulates in *xrn4*. Examples of decapped intermediates accumulating in *xrn4* for three other miRNA target transcripts in polyA+ or polyA- RNA (or both) were also identified (e.g. Supplementary Figure S5). We conclude that the 3′ cleavage fragments of miRNA targets that overaccumulate in *xrn4* PARE correspond to XRN4 substrates, and that most are polyadenylated. Further, some miRNA targets were found to be XRN4 substrates on the basis of overaccumulating decapped intermediates in both *xrn4* mutants.

### *xrn4* overaccumulates many decapped decay intermediates of mRNAs, including NMD-sensitive transcripts

Expanding our analysis of XRN4 substrates beyond miRNA targets, we hypothesized that many more mRNA transcripts would be subject to decapping. Supportive data came from mapping the 5′ end of the most abundant PARE sequence on a transcript from each library (referred to as the ‘MaxSeq’) to one of four delineated regions (100 nt upstream, 5′UTR, CDS and 3′UTR), and repeating this for all protein-coding transcripts (Supplementary Figure S6A). The transcripts were modified to include 100 nt upstream of the annotated transcription start site (TSS) to counter the possibility of inaccurate annotation of the cap position. Two key observations were apparent. First, in polyA+ and polyA- PARE libraries of WT, the MaxSeqs were largely distributed across the CDS region, whereas in the polyA+ and polyA- libraries of *xrn4–5* and *xrn4–6*, the proportion of MaxSeqs in the 5′UTR and the 100 nt upstream regions were higher. Second, this increase was largely due to that of the 5′ UTR (which includes the annotated cap site/TSS) and was more prominent in polyA- PARE. Although PARE libraries capture decay intermediates across the length of the transcript, this biased distribution of MaxSeqs in *xrn4* mutants suggested that many polyA+ and polyA- mRNA decay intermediates were decapped.

To test this idea, identifying the exact cap sites of Arabidopsis transcripts was necessary. This was accomplished using C-PARE, a variation of PARE developed specifically to capture the 5′ ends of capped RNA after *in vitro* decapping, as described in Supplementary Figure S3B and (30). As was done for the PARE libraries, the C-PARE libraries were matched to the annotated mRNA sequences (which included the 100 nt upstream of the TSS) and C-PARE MaxSeqs were identified. Approximately 7200 transcripts had cap sites within +25 nt of the annotated cap site and about 2260 mapped precisely to this site (Supplementary Figure S6B, inset). Distribution of cap sites was comparable between WT and *xrn4–5* and, in total, the C-PARE analysis identified 14010 (12358 protein-coding) transcripts, to be used in subsequent analyses.

**Figure 3.**
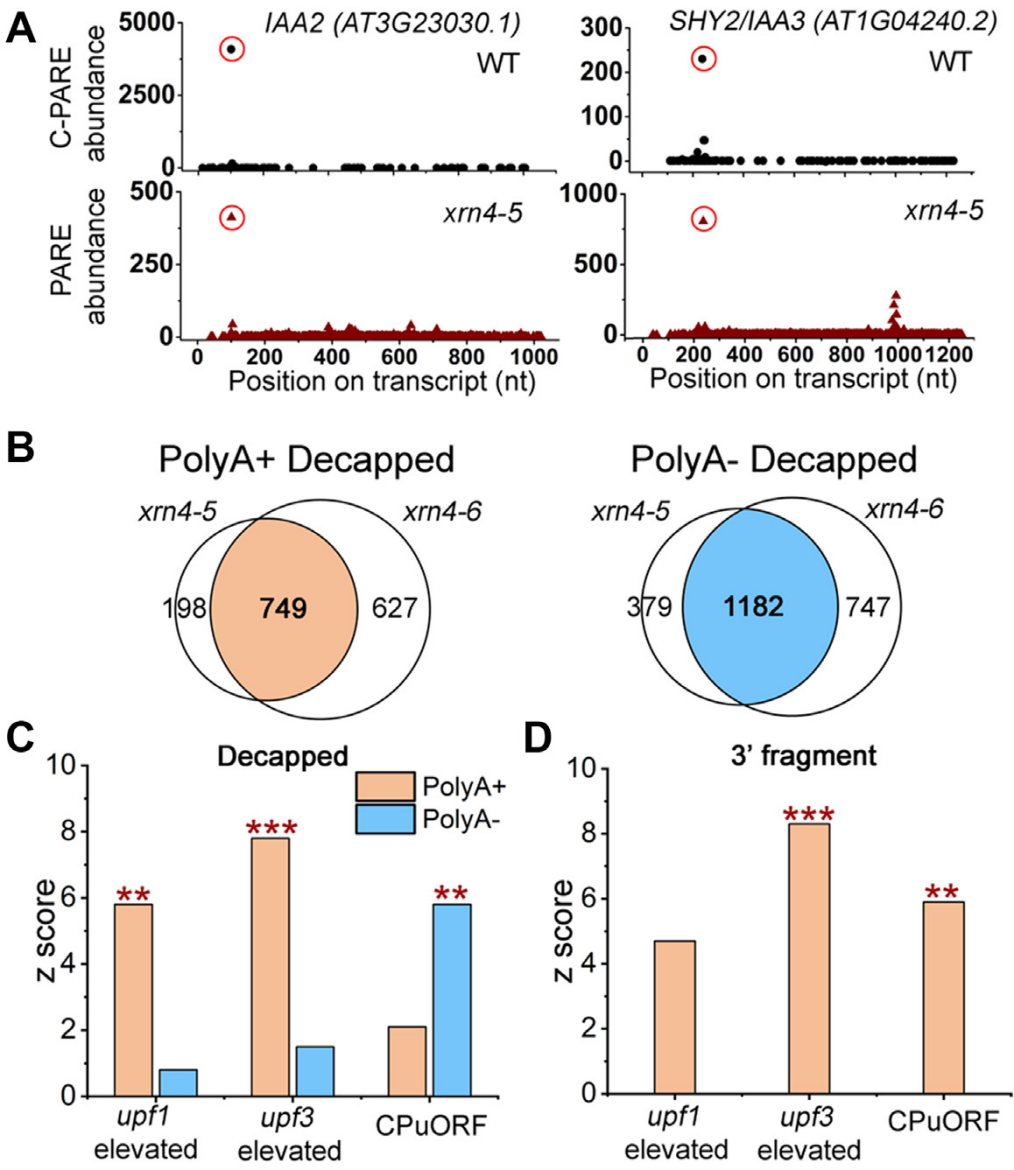
XRN4 substrates include decapped intermediates and 3′ fragments of select RNAs. (**A**) D-plots of *IAA2* and *SHY2/IAA3* mRNAs show profiles from C-PARE (WT, top) and polyA- PARE (*xrn4–5*, bottom). Red open circles indicate the cap sites (top) and polyA- MaxSeqs that precisely coincide with cap sites (bottom). (**B**) Weighted Venn diagrams show overlap between *xrn4–5* and *xrn4–6*, of elevated MaxSeqs that match exactly to cap sites and represent decapped intermediates from unique transcripts in polyA+ or polyA- PARE libraries. The overlap between the two mutants corresponds to decapped XRN4 substrates resulting from the pipeline in Supplementary Figure S7. (**C**) Overlap between NMD-sensitive transcripts and decapped XRN4 substrates. (**D**) Overlap between NMD-sensitive transcripts and 3′ fragments overaccumulating in *xrn4* polyA+ PARE. For (C and D), CPuORFs, and *upf1*- and *upf3*-elevated datasets are described in Supplementary Table S4. *Y*-axis, *z*-scores were generated by the GeneSect program and significant overlaps between datasets are indicated by *P*-values (**, *P*< 0.01; ***, *P* < 0.001). For panel (D), in both *xrn4* mutants MaxSeq at the same site, abundance ≥ 20 TP20M and ≥2 fold change as described in Supplementary Experimental Procedures.

Using the cap site information, the MaxSeqs overaccumulating in polyA+ and polyA- *xrn4* PARE libraries were analyzed as per the schema in Supplementary Figure S7. The 5′ ends of MaxSeq positions that coincided exactly with cap sites (e.g. Figure 3A), met minimal abundance criteria (≥10 TP20M in *xrn4*), and were ≥5 fold more abundant in *xrn4* than WT were considered to represent decapped decay intermediates of the corresponding transcripts (Figure 3B and Supplementary Dataset S2). Transcripts with decapped intermediates fulfilling these criteria and were common to *xrn4–5* and *xrn4–6* were designated as decapped XRN4 substrates because such intermediates are exactly those that would overaccumulate in the absence of the enzyme. The number of decapped XRN4 substrates in polyA- (1182 transcripts) was about 1.5 times higher that of those in polyA+ (749 transcripts), reflecting a trend observed for decapped intermediates in each of the individual *xrn4* mutants (Figure 3B).

This would predict that the decay of some XRN4 substrates would be sensitive to inhibition of decapping. We evaluated this with data from a recent study that investigated RNA decay on a global scale in the decapping mutant, *vcs-7* (79). In that study, using a new computational approach, mRNA *t*_1/2s_ were estimated by choosing the best among a large number of decay models for each transcript. A majority (67% of polyA+ and 70% of polyA-) of XRN4 substrates with *t*_1/2s_ ≤ 240 min showed ≥1.5 times longer RNA *t*_1/2_ when decapping was defective (Supplementary Figure S8 and Supplementary Dataset S2). As would be expected, these data confirm that the decay of many XRN4 substrates are impacted by VCS and fit well with our PARE analysis that demonstrated the importance of decapping and 5′ to 3′ decay.

One category of transcripts that overaccumulates decapped intermediates in *xrn4* is comprised of those with annotated CPuORFs in their 5′ UTRs. The CPuORFs encode peptides which, in multiple cases, have been shown to translationally control the downstream ORF (80–83). Upstream ORFs (uORFs) are one of the features that can trigger NMD, which fits with the observation that CPuORF-containing transcripts were highly over-represented among the transcripts elevated in NMD mutants (31,32). Among the 47 CPuORF-containing transcripts with cap site information from C-PARE, 18 were found among decapped XRN4 substrates from polyA+ and/or polyA- libraries (Supplementary Dataset S2). We utilized the GeneSect tool (on the Virtual Plant v1.3) that is based on Monte Carlo testing to measure the probability that two datasets overlap by more than expected due to chance. Our results indicate a significant overlap (*z*-score > 5) between CPuORF-containing transcripts and decapped XRN4 among polyA- substrates (Figure 3C).

To expand on other NMD-sensitive transcripts that are XRN4 substrates (Figure 3C), transcripts elevated in key NMD defective mutants, *upf1* and *upf3*, were also compared. A recently published microarray study was used that employed growth conditions very similar to our work (32). Cap site information was available for about 33% of the *upf*-elevated transcripts (Supplementary Table S4). A total of 63 and 94 transcripts elevated in *upf1* and *upf3*, respectively, were distributed among decapped polyA+ and/or polyA- XRN4 substrates (Supplementary Dataset S2). Interestingly, a larger proportion of decapped XRN4 substrates from polyA+ were elevated in *upf* mutants than those in polyA- (Figure 3C). For example, of the decapped XRN4 substrates 68 out of 749 polyA+ (9.1%) and 44 out of 1182 polyA- (3.7%) transcripts corresponded to those elevated in *upf3* (Supplementary Dataset S2 and Supplementary Table S4). GeneSect further confirmed a significant overlap between *upf*-elevated transcripts and decapped XRN4 substrates that are polyA+ and not polyA- (Figure 3C). These results for *upf*-elevated transcripts was clearly opposite from those of the CPuORF-containing transcripts (Figure 3C). This difference in distribution of decay intermediates suggests that XRN4 substrates that are CPuORF-containing transcripts are more likely to be deadenylated prior to XRN4-mediated decay, whereas those from *upf1*- and *upf3-*elevated transcripts are not.

### *xrn4* overaccumulates 3′ fragments of NMD-sensitive transcripts

MaxSeqs in the coding region or 3′ UTR that did not match cap sites (3′ fragments) were also investigated (Figure 3D). This analysis was restricted to polyA+ PARE libraries because very few 3′ fragments were detected in polyA- PARE (Figure 2B). A total of 568 3′ fragments overaccumulate (≥2 fold) at the same site in both *xrn4* mutants compared to the WT. We found that 37 and 63 of these sites correspond to transcripts elevated in *upf1* and *upf3*, respectively. GeneSect indicated significant overlap between *upf3*-elevated transcripts and 3′ fragments overaccumulating in *xrn4* (Figure 3D). However, *upf1*-elevated transcripts showed only a modest association with 3′ fragments (Supplementary Table S4) and is likely because NMD is not completely inhibited in the hypomorphic *upf1–5* mutant used in the study (32,84). Among the CPuORFs, nine transcripts overaccumulated 3′ fragments in *xrn4* and this overlap between the two datasets was found to be significant (Figure 3D). These results suggest that there is a higher incidence of 3′ fragment accumulation among NMD-sensitive transcripts in Arabidopsis.

**Figure 4.**
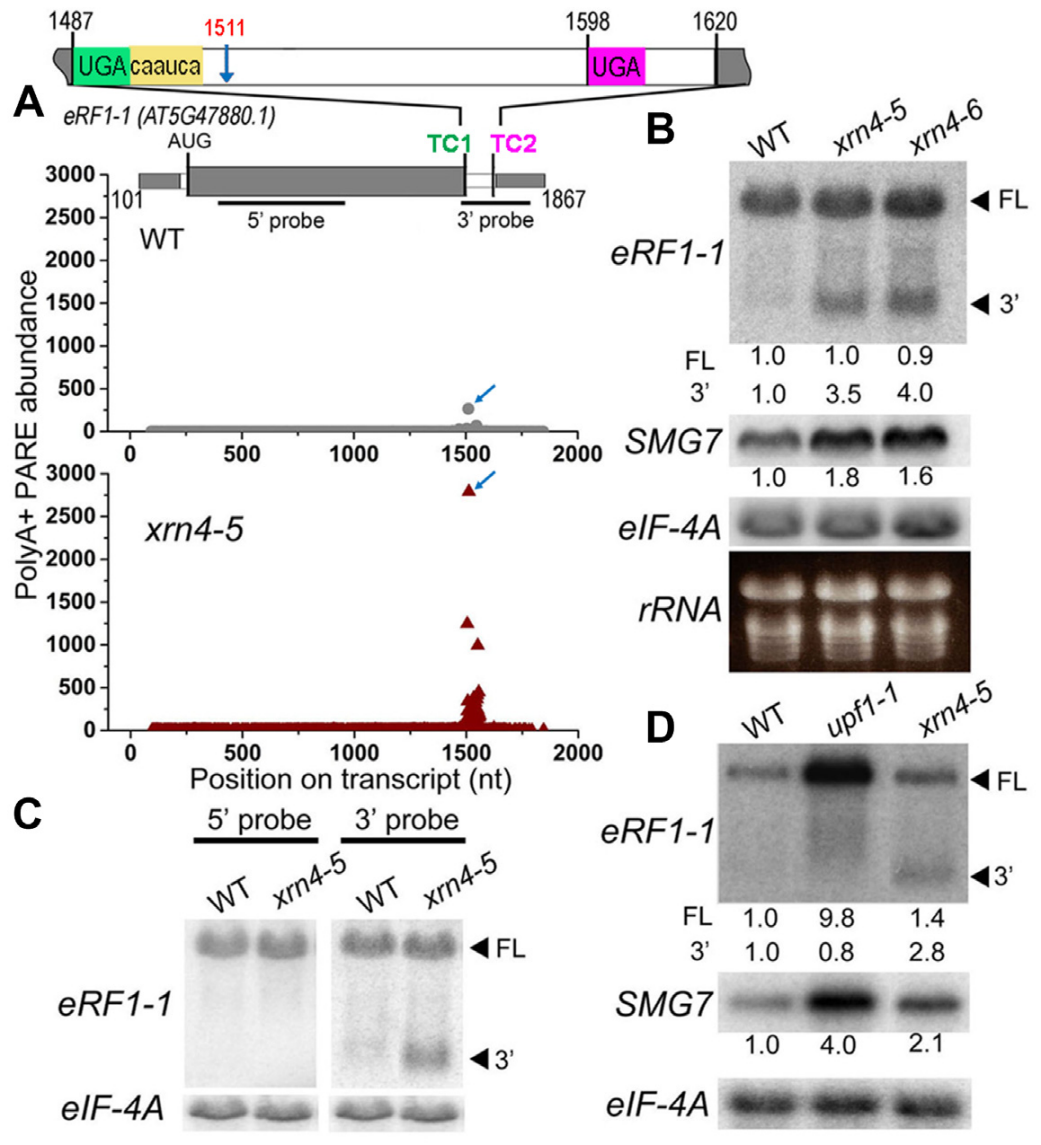
*xrn4* mutants show enhanced accumulation of 3′ fragment of an NMD target RNA, *eRF1–1*. (**A**) D-plots show prominent 3′ fragment of *eRF1–1* RNA in WT and *xrn4–5* polyA+ PARE. Blue arrow, MaxSeq in *xrn4* and its corresponding position (1511 nt) in WT. Structure of *eRF1–1* mRNA: exons in alternating gray and white box; TC1 and TC2, stop codons; yellow, read through element. Black bars, position of 5′ and 3′ probes used in northern blots. (**B**) RNA levels of full-length (FL) and 3′ fragment (3′) of *eRF1–1* in WT, *xrn4–5* and *xrn4–6* seedlings. (**C**) Detection of *eRF1–1* 3′ fragment in WT, and *xrn4–5* seedlings by either 5′ (left) or 3′ (right) probe (described in A). (**D**) Levels of *eRF1–1* RNA in WT, *upf1–1* and *xrn4–5* flowers. RNA levels of NMD marker, *SMG7* are also presented (B and D). Northern blots are from total (B and C) or polyA+ (D) RNA samples. Values indicate fold-changes normalized to *eIF-4A* levels and WT abundance set to 1.

One of these 3′ fragments in a known NMD factor and target, *Eukaryotic Release Factor 1–1* (*eRF1–1*) mRNA (31,32,85), was examined in more detail. The D-plot identified a highly abundant 3′ fragment beginning in the 3′UTR that overaccumulated in *xrn4* polyA+ PARE (Figure 4A). The MaxSeq was located 22 nt downstream of the annotated stop codon (TC1) of *eRF1–1* mRNA and was 10-fold higher in *xrn4* than WT. C-PARE analysis of *eRF1–1* did not show any PARE sequence in and around this position, arguing against the possibility of an internal cap site (data not shown). Northern analysis with a 3′ probe detected the full-length (1.7 kb) *eRF1–1* and a short (about 0.5 kb) RNA corresponding to a prominent 3′ fragment (3′) overaccumulating in *xrn4–5* and *xrn4–6* but barely visible in WT (Figure 4A and B). This 3′ fragment does not hybridize with a 5′ probe that was upstream of the MaxSeq (Figure 4C), nor does it accumulate in the *xrn2–1xrn3–3* double mutant which is deficient in the nuclear XRNs (Supplementary Figure S9B). 5′ RACE analysis showed that the 5′ end of major 3′ fragment overaccumulating in *xrn4–5* matches the MaxSeq position, indicating that the blot and the PARE data are reporting on the same 3′ fragment (Supplementary Figure S9A).

*eRF1–1* mRNA has characteristic features that trigger NMD in plants, such as a long 3′ UTR (>350 nt) with an intron located >50 nt downstream of the stop codon (Figure 4A, TC1) (37,86). Recently, *eRF1–1* was shown to undergo autoregulation by a unique translational readthrough (RT)-NMD mechanism (87). *eRF1–1* has an RT element (CARYYA consensus) next to TC1 in addition to a downstream stop, TC2, (Figure 4A) near the polyadenylation signal in the 3′ UTR. NMD was found to be induced by the 3′ UTR of *eRF1–1* and the presence of the RT only partially rescued the *eRF1–1* mRNA from NMD (87). Our results indicate that the 3′ UTR fragment of *eRF1–1* is cleaved downstream of TC1, and this is one of the mechanisms for the turnover of *eRF1–1*. This fits with the metazoan model for NMD, whereby UPF1-dependent SMG6 cleavage occurs near the PTC that triggers this initiating degradative event (29,30).

To examine sensitivity of the *eRF1–1* transcripts to NMD, its mRNA levels were analyzed in a well-studied missense mutant that leads to a dysfunctional UPF1 (61), *upf1–1* using *SMG7* mRNA as a positive control (Figure 4D). SMG7 is an NMD factor encoded by an NMD-sensitive mRNA in plants and other systems (21,31,33,88,89); its mRNA levels are elevated in *upf1* as expected (31,33,61) and to a modest extent in *xrn4* mutants (Figure 4B and D; Supplementary Figure S9C). Full-length *eRF1–1* mRNA was considerably elevated in *upf1–1* (Figure 4D) and after treatment with the translation inhibitor cycloheximide (CHX), which is known to inhibit NMD (Supplementary Figure S9C). Moreover, the 3′ fragment of *eRF1–1* observed in *xrn4–5* was absent in *upf1–1* and following CHX treatment. This is consistent with 3′ fragment of *eRF1* mRNA being NMD sensitive. Whereas XRN1-dependent NMD-sensitive 3′ fragments are well documented for many NMD targets in animal cells (27–30), these observations point towards a novel post-transcriptional mechanism for *eRF1–1* turnover in plants.

### *xrn4* overaccumulates decapped intermediates of transcripts involved in photosynthesis, hormone and abiotic stress responses

**Figure 5.**
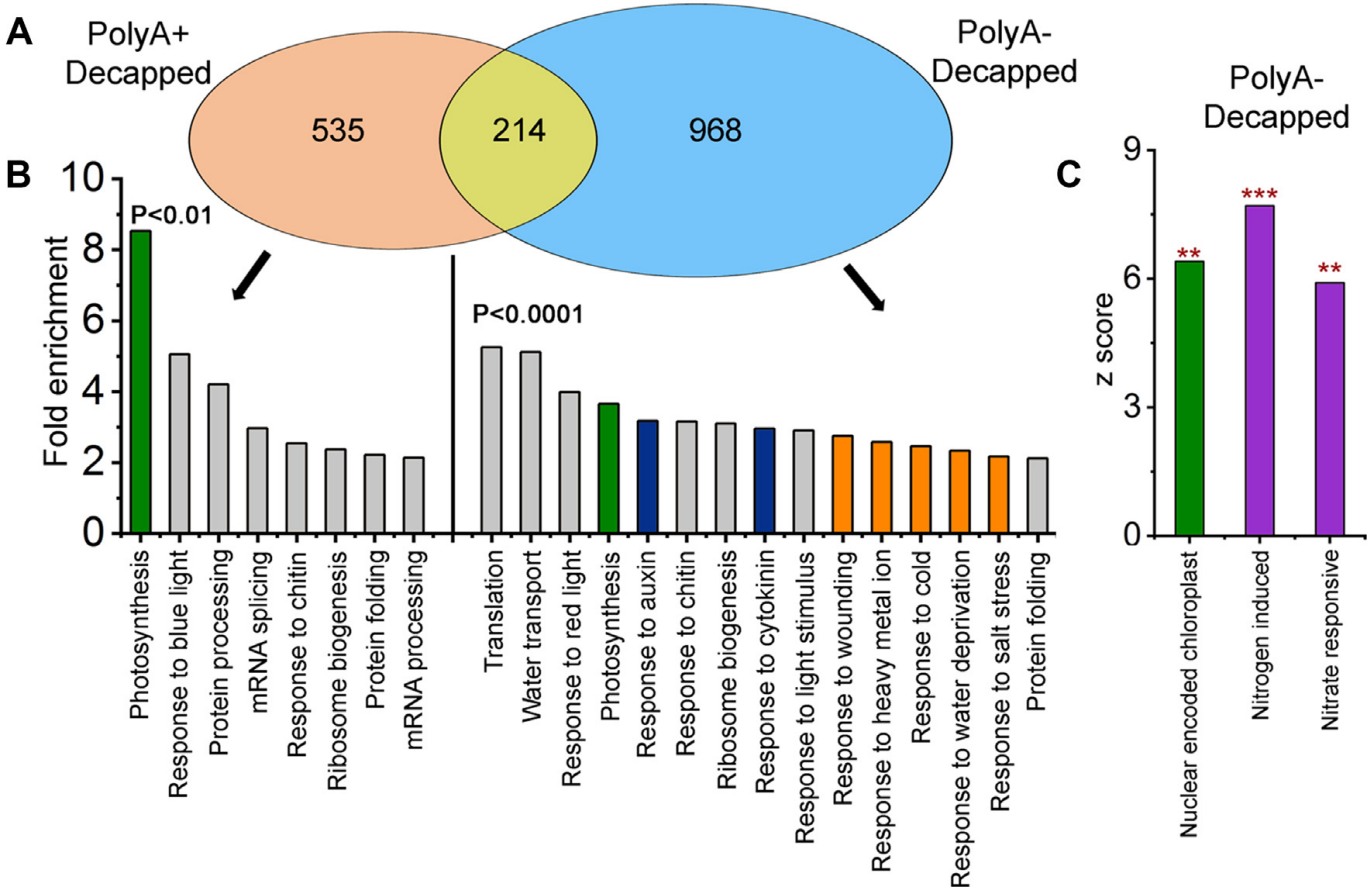
Decapped XRN4 substrates are over-represented for categories associated with photosynthesis, stress and hormone responses. (**A**) Transcripts overaccumulating decapped intermediates in both *xrn4–5* and *xrn4–6* were identified from the overlap data in Figure 3B. Weighted Venn diagrams designate these as decapped polyA+ (polyadenylated) and polyA- (deadenylated) XRN4 substrates as discussed in the text. (**B**) Gene-ontology (GO) enrichment categories of decapped XRN4 substrates. Highlighted are significant GO functions associated with photosynthesis (green), abiotic stimulus/responses (orange), hormone responses (blue) and other categories (gray). Only 15 most significant terms are presented for polyA- and the rest are listed in Supplementary Table S5 (PARE). (**C**) Nitrogen (N) responsive and NEC transcripts are over-represented among XRN4 substrates. Overlap significance between decapped XRN4 substrates (**, *P* < 0.01; ***, *P* < 0.001) and transcripts from different datasets was evaluated using the GeneSect program (Supplementary Table S4).

The impact of XRN4 on processes of biological significance beyond NMD was investigated on the basis of its decapped substrates identified from polyA+ PARE, polyA- PARE or both (Figure 5A and B; Supplementary Figure S10). Gene-ontology (GO) annotations for biological processes that were enriched among XRN4 substrates were identified (Figure 5B). In particular, the deadenylated substrates included a diverse array of categories that prompted a cut-off of *P* < 0.01 to select those with the most potential significance (PARE, Supplementary Table S5). GO annotations associated with photosynthesis, hormone responses, ribosome biogenesis, protein folding and response to chitin were highly over-represented among both polyadenylated and deadenylated substrates. In addition, polyadenylated substrates included processes such as splicing and response to blue light. In contrast, the deadenylated substrates were associated with light stimuli, water transport, response to auxin and cytokinin and different abiotic stress responses (metal ion, cold, salt and osmotic). This suggests that some of the mRNAs associated with stress and hormone responses preferentially undergo deadenylation prior to XRN4-mediated decay.

PARE analysis was also expanded to examine the overlap between decapped XRN4 substrates and other related datasets, and the significance of the commonality was tested using the GeneSect tool. The intersection between XRN4 substrates and nuclear-encoded chloroplast (NEC) genes was also examined. NEC genes include those encoding critical components of the photosynthetic machinery that are central to chloroplast function (90,91). A significant overlap was observed for NEC genes and is consistent with the finding that gene products associated with photosynthesis and light responses were the most highly enriched GO categories among decapped polyA+ and polyA- XRN4 substrates (Figure 5B). Examples of decapped RNA intermediates that overaccumulate strongly in *xrn4* mutants include *LHCB1, LHCB3, CAB2* and *RBCS1A* (Supplementary Dataset S2). In agreement with our results, an earlier study, which examined a combination of tilling arrays, microarrays and PARE data from *xrn4–5* flowers, also reported an enrichment of NEC transcripts in the mutant (46). That decapped intermediates of photosynthetic transcripts overaccumulate as polyadenylated or deadenylated transcripts indicate that multiple mechanisms involving XRN4 are responsible for their decay.

### *xrn4* exhibits enhanced dark-induced chlorophyll loss of leaves and seedlings

**Figure 6.**
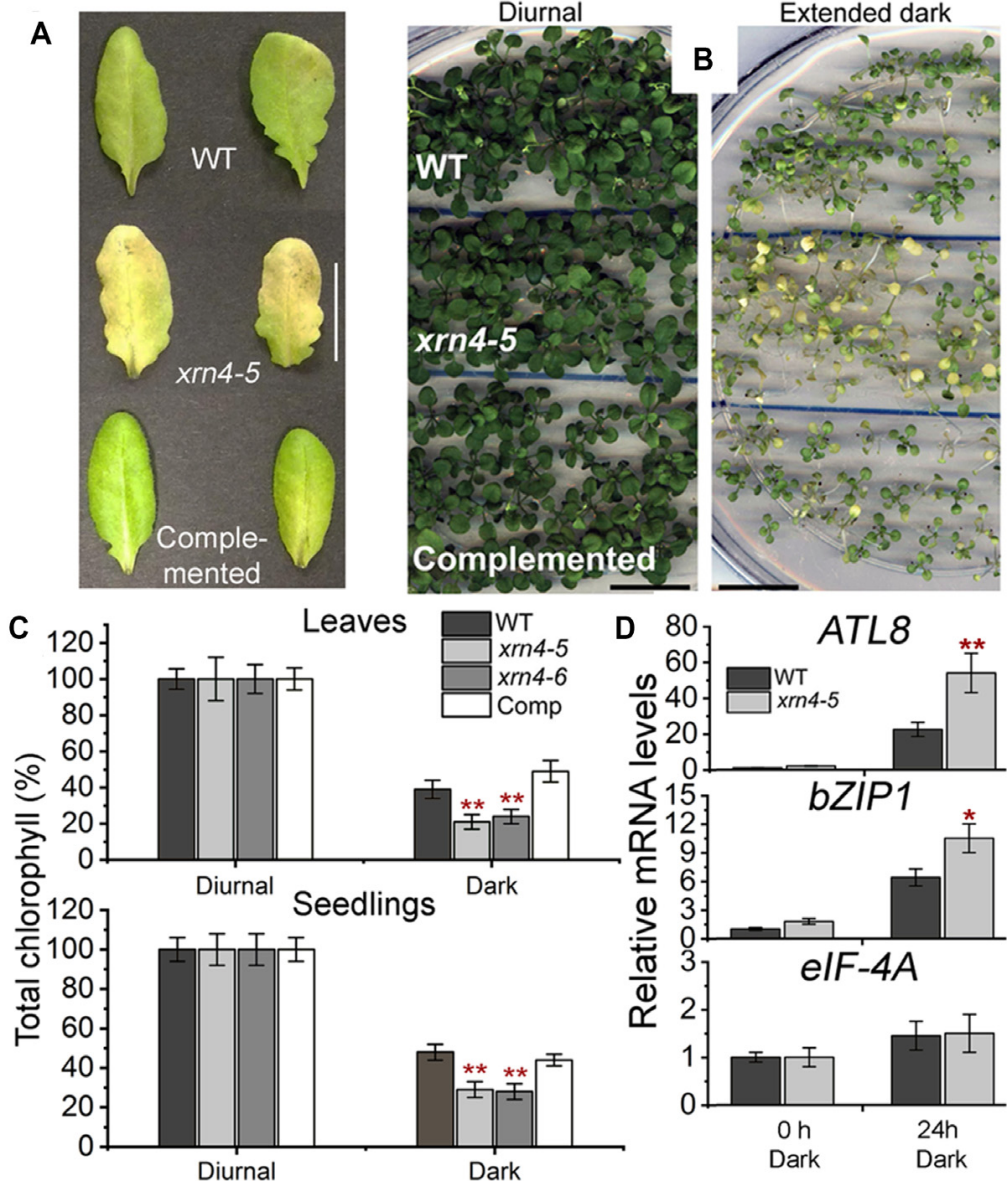
*xrn4* is oversensitive to dark treatment of leaves and seedlings. Detached leaves (**A**) and intact seedlings (**B**) of WT, *xrn4–5* and complemented line of *xrn4–5* (Comp) after extended dark (A, 4 d and B, 12 d) treatments; scale bar, 20 mm. (**C**) Chlorophyll content of dark treated (leaves, 4 d and seedlings, 12 d) WT, *xrn4–5, xrn4–6* and Comp presented as a percentage of diurnal (100%). Histograms are means from three biological replicates; four leaves from a total of 12 plants per genotype per condition (A) and a total of 15 pools of seedlings (5 to 6 plants per pool) per genotype per condition (B) were assayed and presented as percentages ± standard error of the mean (SEM). (**D**) Quantitative RT-PCR of polyA+ RNA show mRNA levels of *ATL8, bZIP1* and *eIF-4A* (control transcript) in 2-week-old seedlings of WT and *xrn4*–*5* after 0 h and 24 h of dark treatment. WT levels at 0 h set to 1. Data are means ± standard deviation (SD) from four biological replicates; **, *P*< 0.01 and *, *P*< 0.05.

Given the role of XRN4 in the turnover of mRNAs from genes involved in photosynthesis, a related biological deficiency in plants lacking the enzyme was sought. Our rationale was that atypical growth conditions such as extended darkness might exacerbate any impaired photosynthetic function in *xrn4*. To test this, *xrn4–5, xrn4–5* complemented with a genomic copy of XRN4 (with its native promoter and terminator), and WT were first grown under normal photoperiod, and then whole seedlings, or detached fully expanded rosette leaves were transferred to darkness (Figure 6A and B). Dark-treated seedlings or detached leaves showed significant decreases in total chlorophyll content compared to the photoperiod controls (referred to as ‘Diurnal’). Dark-treated leaves of *xrn4* mutants showed significant decreases (40–50%) in total chlorophyll content and exhibited severe chlorosis, compared to the dark-treated WT and complemented line (Figure 6C). Even at the whole-plant level, the dark-treated *xrn4–5* seedlings clearly showed severe chlorosis, exhibiting a 30–40% decrease in total chlorophyll content compared to the dark-treated WT and the complemented line (Figure 6C). These results indicate that dark-induced degradation of chlorophyll is more rapid in *xrn4* than WT and stable transformation of the mutant with a genomic copy of *XRN4* is sufficient to correct this deficiency.

Finding that transcripts responding to carbohydrates were among XRN4 substrates, we examined polyadenylated mRNA levels of sugar-starvation responsive marker genes, *bZIP1* (92) and *ATL8* (93), in dark-treated seedlings (Figure 6D). As expected, a 24-h dark treatment strongly elevated the levels of polyadenylated *bZIP1* (>6.5-fold) and *ATL8* (>22-fold) mRNAs in the WT, as opposed to control plants. Levels of these marker mRNAs were even higher in dark-treated *xrn4–5*, especially for *ATL8*, indicating that the mutant plants are much more starved for photosynthates. Together, our results clearly demonstrate that XRN4 is required for normal responses to dark stress.

### *xrn4* mutants exhibit a lateral root (LR) defect during N resupply

**Figure 7.**
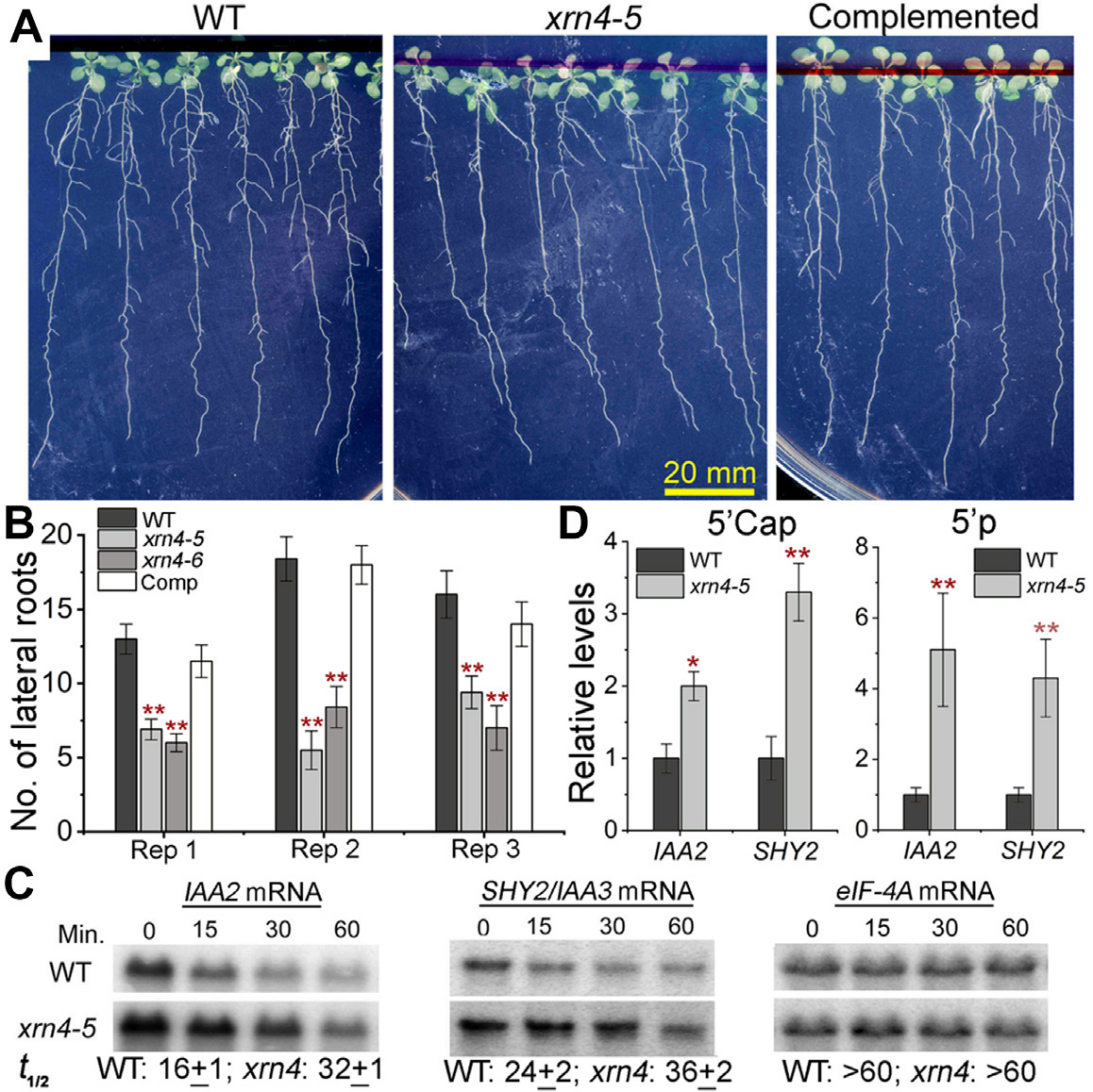
*xrn4* mutants show attenuated lateral root growth after recovery from N starvation. (**A**) Root images showing LR phenotype of WT, *xrn4–5* and its complemented line after 7 d of recovery from 7 d of N starvation. (**B**) Number of LR for WT and *xrn4* mutants from three independent experiments are shown (*N* = 12, 26 and 15 seedlings per genotype). Data are means ± SEM. (**C**) RNA half-life (*t*_1/2_) of *IAA2* and *SHY2/IAA3* under control conditions. Total RNA northern blots show time course of RNA abundance in WT and *xrn4–5* after cordycepin treatment. Average *t*_1/2_ in min ± SD of two biological replicates is below. RNA levels of stable transcript *eIF-4A* are also shown. (**D**) Quantitative splinted-ligation RT-PCR of total RNA shows levels of decapped (5′-P) and capped (5′ Cap, CIP + TAP) RNAs of *IAA2* and *SHY2/IAA3* in WT and *xrn4–5* seedlings. Levels of control RNAs are in Supplementary Figure S13. Histograms are means ± SD of four biological replicates; **, *P* < 0.01 and *, *P* < 0.05.

To examine a novel potential association of XRN4, the overlap between decapped XRN4 substrates and two different nitrogen (N)-responsive transcriptomic datasets were tested (Figure 5C). This was prompted by the initial observation of N-metabolism genes enriched among *xrn4*-elevated transcripts (RNA-seq, Supplementary Table S5). Both N-responsive datasets showed significant overlap with decapped XRN4 substrates, suggesting a new connection between XRN4 and N responses (Figure 5C and Supplementary Table S4). We hypothesized that *xrn4* may be deficient in responding to different N regimes. This was investigated by transferring *xrn4* and WT seedlings to N-starvation and N-replete medium for 7 d and then moving each seedling set to N-replete medium for 7 d (Figure 7A). Strikingly, both *xrn4–5* and *xrn4–6* seedlings that were transferred from N-starvation to N-replete media (N resupply conditions) displayed dramatic reductions (between 50% and 60%) in the overall number of LR compared to the WT and the complemented line of *xrn4–5* (Figure 7A and B) across multiple biological replicates. In contrast, all nonstressed seedlings (WT and mutants) on N-replete medium throughout the experiment (control conditions) showed a higher and comparable number of LRs (Supplementary Figure S11A). Also, both *xrn4* mutants did not show any apparent LR defects under control conditions, or N-starvation conditions. Under the N resupply conditions, no significant differences in primary root elongation were observed for *xrn4* mutants compared to WT and the complemented line (Supplementary Figure S11B). These results demonstrate that XRN4 is required for normal LR growth during N resupply.

### *xrn4* mutants impact mRNA levels of *AUX/IAA* and *SAUR* genes

Several studies have implicated auxin as a major player in modulating root proliferation during plant responses to N (94–99). As presented in Figure 5B, the GO analysis showed a large number of auxin-responsive transcripts among deadenylated and decapped XRN4 substrates. We found several auxin-responsive transcripts belonging to *AUX/IAA* (7) and *SAUR* (14) gene families strongly overaccumulated decapped intermediates in *xrn4* polyA- PARE (Supplementary Figure S12A). The abundances of these transcripts were also elevated in *xrn4* mutants in the polyA- RNA-seq data with an average fold change of ≥2, indicating that these mRNAs prefer to undergo deadenylation prior to 5′ to 3′ decay.

Among the *AUX/IAA* family members, *IAA2* previously identified as a Gene with Unstable Transcript (GUT, (100)), and *SHY2/IAA3* strongly overaccumulated decapped intermediates in *xrn4* mutants in polyA- PARE and total RNA, with a modest increase in capped RNA in the latter (Figures 3A and 7D; Supplementary Dataset S2). Therefore, we tested whether the higher abundance of these transcripts was due to increased RNA stability in the mutant (Figure 7C). RNA *t*_1/2_ assays were carried out by inhibiting transcription using cordycepin and monitoring RNA abundance as a function of time. In WT, *IAA2* showed a very short *t*_1/2_ of 16 min, corroborating previous work establishing it as an unstable transcript (100), and *SHY2/IAA3* similarly showed a short *t*_1/2_ of 24 min (Figure 7C). Both *IAA2* and *SHY2/IAA3* mRNAs were more stable in the absence of XRN4 with a t_1/2_ of > 30 min (Figure 7C). The multi-model RNA decay study mentioned earlier (79) estimated RNA *t*_1/2_ of *IAA2* and *SHY2/IAA3* in Col-0 to be 23 and 20 min, respectively, very close to our measured values. Further, these two *AUX/IAA* transcripts are 5′ to 3′ decay substrates as they exhibit longer *t*_1/2_ (>45 min) in *vcs-7* (Supplementary Dataset S2). Therefore, our results show that XRN4-mediated decay contributes to the rapid decay of *IAA2, SHY2/IAA3* and possibly other *AUX/IAA* family members.

## DISCUSSION

A major goal of this work was to investigate the impact of XRN4 on processes of biological significance, which were postulated to be impacted, based on substrates of the enzyme. Using a multifaceted RNA degradome approach, we identified XRN4 substrates that led us to find that the response to dark stress and normal root growth during N resupply are compromised in *xrn4* mutants. Our research also enhanced understanding of RNA decay pathways involving XRN4, most notably NMD. Clear evidence for multiple decay pathways of NMD targets was found, including decay involving internal mRNA cleavage. Because of the absence of SMG6 in plants, this argues for a novel mechanism initiating the decay of some NMD targets that are XRN4 substrates.

This study differed from those carried out previously by examining the polyA- transcriptome separately from polyA+ in *xrn4*. That this would be fruitful was first apparent with RNA-seq, which identified several hundred deadenylated transcripts that were significantly elevated in the absence of *XRN4* (Figure 1 and Supplementary Dataset S1). The observed modest impact of *xrn4* on the polyA+ transcriptome was as expected based on prior work (12,16,44–46) and these transcripts are not necessarily substrates and their elevation could be due to indirect effects of XRN4 loss. Using cap site information and PARE, we demonstrated that *xrn4* overaccumulates decapped RNAs, particularly those that are deadenylated (Figure 3B). The latter results are precisely what is expected for transcripts degraded via deadenylation-dependent decapping, a mechanism that we conclude is degrading many XRN4 substrates in plants.

Not all XRN4 substrates are deadenylated, and mRNAs targeted for AGO cleavage by miRNAs are prominent examples. For those overaccumulating 3′ fragments in *xrn4*, 77% did so in polyA+, compared to 23% in polyA- (Figure 2B and Supplementary Table S3). Since most of the 3′ fragments found in polyA- were also overaccumulating in polyA+, they may result from deadenylation after miRNA-directed cleavage. Decapping of miRNA targets has also been observed for several XRN4 substrates and, in most cases, both decapped transcripts and 3′ fragments are evident in polyA+ (e.g. Supplementary Figures S4C and S5). *AP2* mRNA is among these examples but, in polyA-, it only overaccumulates decapped mRNA in *xrn4* (Supplementary Figure S5A). These data demonstrate that cleavage, decapping and/or deadenylation are all routes of miRNA target turnover prior to degradation by XRN4.

Among the major decapped substrates of XRN4 are genes involved in photosynthesis (Figure 5). XRN4′s role in photosynthetic function is evidenced by enhanced sensitivity of *xrn4* seedlings and detached leaves to extended darkness, indicated by leaves that were severely chlorotic compared to WT and the XRN4 complemented line (Figure 6A, B and C). Multiple impairments are known to result in dark-stress induced leaf chlorosis, including defects in autophagy, electron transport and the low energy response (LER) pathway. The possibility that *xrn4* mutants are defective in the LER pathway is particularly intriguing.

In agreement with a role of XRN4 in the LER, mRNA levels of two well-known sugar-responsive marker genes, *ATL8* and *bZIP1*, were elevated to a higher extent in *xrn4* compared to the WT in response to 24-h dark treatment (Figure 6D). *ATL8* mRNA levels are tightly correlated with low internal carbon levels after an extended night (93,101). The LER pathway responds to changes in energy, such as elevated carbon levels, which repress the expression of bZIP transcription factors. These include bZIP1 and bZIP53 whose mRNAs are XRN4 substrates (Supplementary Dataset S2). Overexpression of *bZIP1* and *bZIP53* is known to cause accelerated leaf yellowing and dark sensitivity in whole plants, also referred to as dark-accelerated starvation/senescence (92). Therefore, a model where increased bZIP expression in *xrn4* mutants leads to dampening of the LER could provide a simple explanation for the dark sensitivity and chlorophyll loss that we uncovered following prolonged darkness.

Given the complexity of the processes that can lead to this phenotype, other gene expression changes in *xrn4* also may have contributed. Nevertheless, the data argue against the dark/senescence sensitivity being dependent on the role of XRN4 in ethylene signaling. Ethylene is a positive regulator of leaf senescence (102), and *xrn4* alleles display ethylene insensitivity (16,53). However, unlike other ethylene-insensitive mutants that have been reported to exhibit increased tolerance to dark-accelerated starvation/senescence (*ein2* or *ein3*; (103,104)), *xrn4* mutants show the opposite. They are more sensitive than WT, or the complemented mutant line, to prolonged darkness as intact seedlings or detached leaves (a typical dark-accelerated senescence assay) (Figure 6A, B and C). We, therefore, conclude that ethylene cannot explain the defective behavior of *xrn4* mutants following long dark treatments.

Another major finding from our RNA degradome analysis was the compelling evidence for the impact of XRN4 on N responses in Arabidopsis. N starvation and resupply affect a large fraction of the Arabidopsis transcriptome (95,105–108). Our analysis showed significant overlap between N-responsive transcripts (106,109) and decapped deadenylated XRN4 substrates (Figure 5C; Supplementary Table S4). Further, among a set of 93 transcription factors that are differentially regulated within 30 min of N resupply (107), 19 overaccumulate decapped deadenylated intermediates in *xrn4* (Supplementary Table S4). This indicates that rapid changes to the transcriptome could also entail RNA turnover mediated by XRN4 for these transcripts. Evidence of our findings is supplied by *xrn4ski2* double mutants with dysfunctional 5′ to 3′ and 3′ to 5′ cytoplasmic RNA decay pathways that strongly impact the mRNA levels of many N responsive genes (48). These double mutants overproduced anthocyanin in the leaves and displayed stunted growth, both of which were rescued by N fertilization. In our study, *xrn4* mutants show attenuated response toward the stimulatory effects of N resupply and produce significantly fewer (>50%) lateral roots than WT and the complemented line (Figure 7A and B). Our findings indicate that the LR defect is N-resupply specific in *xrn4* plants, since the mutants are unaffected in nonstressed (N-replete) conditions, both in our study, and as noted previously (62).

Auxin has been known for many years to be a major hormone modulating N-responsive LR formation (98), and attenuated lateral rooting is characteristic of mutants impaired in auxin signaling (reviewed in (110)). Support for the XRN4–auxin connection comes from a study that showed reduced auxin-responsive LR formation in *xrn4* (56). Our PARE analysis identified a large proportion of auxin-responsive transcripts, especially *AUX/IAA* and *SAUR* family members that are among decapped XRN4 substrates (Figure 5B and Supplementary Figure S12A). *AUX/IAA* genes code for transcriptional repressors of auxin-regulated gene expression crucial for diverse developmental responses, including LR formation (reviewed in (111,112)). Our work shows that *IAA2* and *SHY2/IAA3* transcripts are short-lived mRNAs and that XRN4-mediated mRNA decay is an important component of this post-transcriptional control (Figure 7C). Therefore, it seems likely that the root branching defect of *xrn4* during N resupply is caused by increased AUX/IAA repressor levels as a result of increased stability of *AUX/IAA* mRNAs. Although normal AUX/IAA-mediated auxin signaling is important for root response to N stimulus, there is limited information regarding the individual roles of AUX/IAA proteins. Transcriptional modules regulated by SHY2/IAA3 and IAA14 control LR emergence and LR initiation/emergence, respectively (99). So far, only IAA14 has been shown to alter the response of N-responsive transcription factor(s) that control LR growth (106), although both IAA14 and SHY2/IAA3 inhibit LR development when overexpressed (113,114). Since *IAA14* and *SHY2/IAA3* are substrates of XRN4 (Figure 7 and Supplementary Dataset S2), it is easy to envision their inefficient repression in *xrn4* during N treatment as shown in the model in Supplementary Figure S12B. In this model, XRN4 rapidly degrades *Aux/IAA* mRNAs, whereas auxin receptors mediate ubiquitination-mediated proteolysis of AUX/IAA repressors in response to N (115). The AUX/IAA mRNA and protein turnover are both important for activation of the auxin-responsive transcriptional cascade to trigger LR development.

How might the changes in AUX/IAA gene expression and LR development that we observed in *xrn4* be explained mechanistically at the RNA level? In multiple systems, there is evidence that decapping activators interact with XRN1 (116–118). In Drosophila, XRN1 directly binds DCP1, a decapping complex protein, and absence of XRN1 leads to less efficient decapping, resulting in increased capped mRNA (116). We observed increased capped and uncapped levels of *SHY2/IAA3* RNA (Figure 7C) and found the RNA to be more stable after blocking transcription in *xrn4* (Figure 7D). This supports the idea that XRN4-coupled decapping could occur during post-transcriptional control of SHY2/IAA3 levels, and thus explain observed LR defect, and potentially other defects, of *xrn4* mutants. In any event, there is certainly precedent for regulatory proteins to have unstable mRNAs and proteins (119). These traits could facilitate rapid changes in gene expression in response to various regulatory stimuli and major biological defects when that control is disrupted.

A key result from our study was to show that XRN4 substrates include NMD-sensitive targets such as CPuORF, and *upf1*- and *upf3*-elevated transcripts (Figure 3C and D; Supplementary Table S4 and Supplementary Dataset S2). Although NMD is a conserved surveillance pathway in eukaryotes, plants lack an ortholog of metazoan SMG6, a PIN-domain containing endoribonuclease. As a result, NMD substrates are thought to predominantly undergo exoribonucleolytic decay from the ends in plants (26). Our work shows that a large percentage (∼38%) of CPuORF-containing mRNAs are turned-over by decapping followed by XRN4-mediated decay (Figure 3C and Supplementary Dataset S2). Interestingly, these decapped CPuORF-containing mRNAs overaccumulate predominantly in the polyA- fraction, suggesting a prominent role for deadenylation. NMD targets are known to undergo deadenylation in yeast and mammals (89,120,121), and our work suggests that it occurs in plants for select XRN4 substrates. Decapped XRN4 substrates include *upf1*- and *upf3*-elevated transcripts, and unlike CPuORF-containing transcripts, majority of them accumulated predominantly in polyA+ (Figure 3C and Supplementary Dataset S2). In yeast, NMD targets generally undergo decapping without requiring deadenylation (122,123). Consistent with this understanding, our work suggests that this mechanism could be conserved in plants.

One of our most intriguing findings was that XRN4 substrates also include 3′ fragments of NMD-sensitive transcripts. PARE analysis identified 62 transcripts that overaccumulate such fragments in *xrn4* (9 CPuORF; 53 *upf1* and/or *upf3*-elevated transcripts Figure 3D; Supplementary Dataset S2). For *eRF1–1* mRNA, we further demonstrate that its 3′ fragment accumulation is nearly undetectable unless XRN4 is deficient (Figure 4; Supplementary Figure S9A, B and C). *eRF1–1* encodes a translation termination release factor that is a component of the SURF (SMG-1-Upf1-eRF1-eRF3) complex (124) and its mRNA has a long 3′UTR with an intron, both typical features that could trigger NMD (87). Our results indicate *eRF1–1* mRNA undergoes endoribonucleolytic cleavage and its decay is dependent on UPF1 and XRN4. Decay of *eRF1–1* initiated by an endoribonucleolytic cleavage should produce 5′ and 3′ fragments that are degraded by the cytoplasmic exosome and XRN4, respectively. Although 5′ fragments are notoriously difficult to detect due to their rapid degradation by 3′ to 5′ exoribonucleases (27,28,125), experiments inactivating the 3′ to 5′ decay pathway indicate their presence. For instance, *eRF1–1* mRNA levels were elevated in *xrn4ski2* compared with *xrn4* and WT (48), supporting the involvement of both 5′ to 3′ and 3′ to 5′ cytoplasmic exoribonucleolytic pathways for turnover of fragments produced by cleavage of *eRF1–1* mRNAs. As in the case of *eRF1–1*, we found other examples in Arabidopsis (Supplementary Figure S9D) that showed a cleavage pattern reminiscent of SMG6 activity observed in mammalian cells (29,30).

Our results implicate an unknown endoribonuclease in the turnover of *eRF1–1* and other selected NMD targets that is distinct from SMG6 seen in metazoans. The Arabidopsis genome codes for several endoribonucleases with a PIN or PIN-like domain (126–129), which could cleave RNAs, leaving a 5′ monophosphate on a 3′ fragment that could be subsequently degraded by XRN4. Plants may have evolved a unique mechanism in the absence of a SMG6 ortholog to cleave NMD targets in the same way they evolved to replace the lack of an XRN1 ortholog with an XRN2 ortholog (XRN4) functioning in the cytoplasm. Further studies are required to identify and characterize the unknown endonuclease and understand the pervasiveness of this mechanism in the plant kingdom. Recently, a PIN-like NYN domain containing putative endoribonuclease was identified among proteins that co-immunoprecipitated with UPF1 (130). It will be exciting to see if this or another PIN-like protein is the plant NMD endoribonuclease cleaving *eRF1* mRNA and other NMD targets with 3′ fragments.

## DATA AVAILABILITY

Dataset containing the cap sites and sequences from C-PARE, raw and processed sequences of PARE and RNA-seq libraries have been deposited at the National Center for Biotechnology Information's Gene Expression Omnibus (GEO) under accession number GSE119706.

## Supplementary Material

gkz712_Supplemental_Files

## Notes

Present address: Patrick M. Kukulich, Sidney Kimmel Medical College, Thomas Jefferson University, Philadelphia, PA 19107, USA.
