# Supplementary material for: RNA degradomes reveal substrates and importance for dark and nitrogen stress responses of Arabidopsis XRN4
Source: Nucleic Acids Res. 2019 Aug 20;47(17):9216–30. doi: 10.1093/nar/gkz712 (PMC6755094; doi:10.1093/nar/gkz712)
Supplement: gkz712_Supplemental_Files [file gkz712_supplemental_files.zip › 072119_Supplemental Figures and Legends_AC.docx]

**Supplementary Material**

accompanying the main manuscript entitled

**RNA degradomes reveal substrates and importance for dark and nitrogen stress responses of Arabidopsis XRN4**

Vinay K. Nagarajan, Patrick M. Kukulich, Bryan von Hagel and Pamela J. Green

Delaware Biotechnology Institute, University of Delaware, Newark, DE 19711

**Table of Contents**

Figures S1 to S13

Tables S1 to S5

Supplementary Experimental Procedures

References

**Supplementary Figures**

**Supplemental Figure S1**


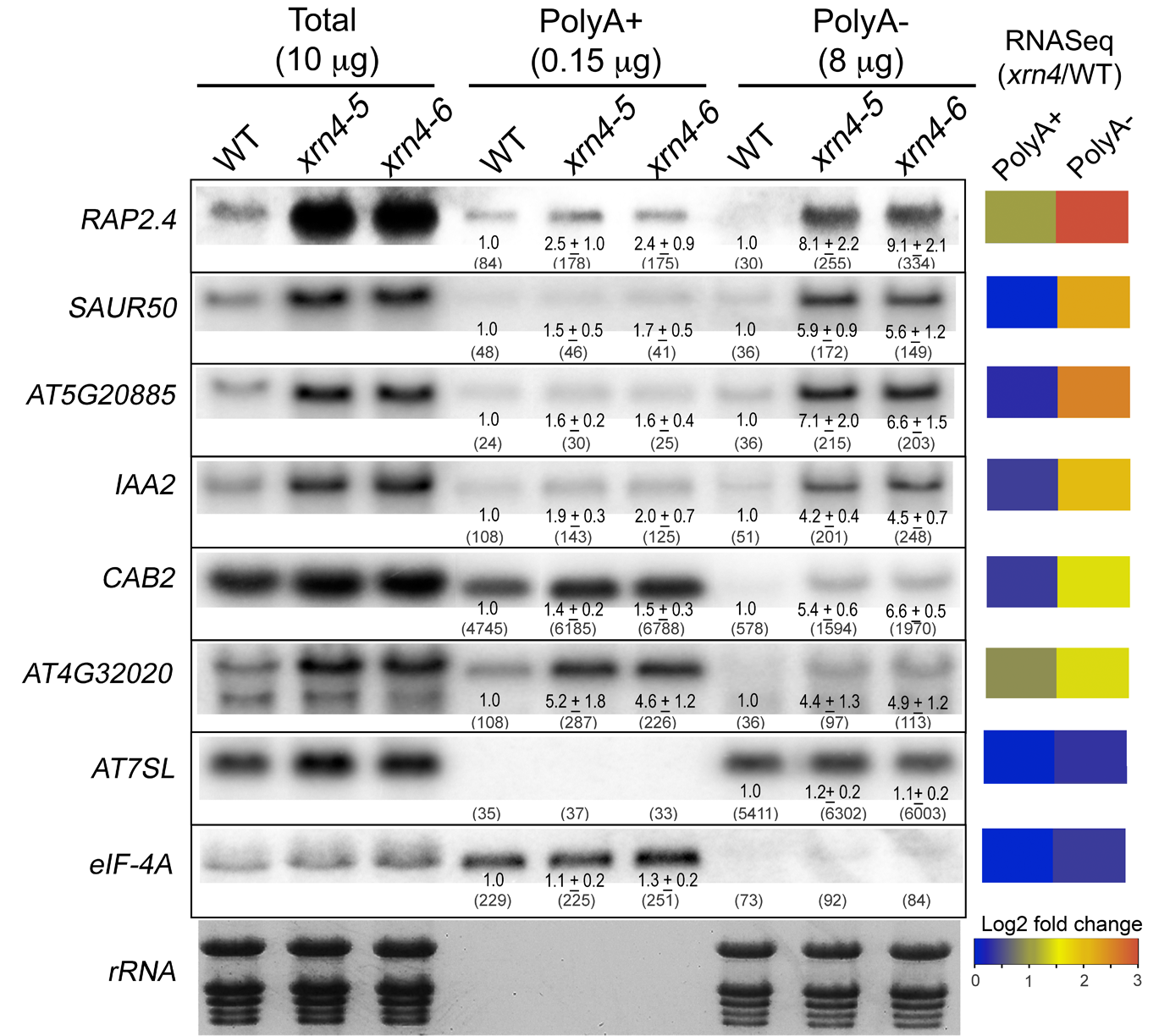


**Supplemental Figure S1. Validation of transcripts elevated in *xrn4* in polyA- RNA fraction.** Northern blots show RNA levels of selected transcripts in polyA+ and polyA- RNA fractions of WT, *xrn4-5* and *xrn4-6* seedlings. Values indicate average fold-changes (+ SD) in *xrn4* mutants from two biological replicates normalized to levels of *eIF-4A* mRNA (polyA+) or *AT7SL* noncoding RNA (polyA-) with WT abundance set to 1. RPKM values from RNA-seq analysis are in parenthesis. Heat map of fold changes (*xrn4-5*/WT) for these transcripts from RNA-seq analyses (Figure 1) are presented on the right. Northern blots were generated based on percent recovery after total RNA fractionation: 1.5% (for polyA+) and 80% (for polyA-) RNA. Fractionated RNA samples were run alongside total input RNA (10 µg) as shown.

**Supplemental Figure S2**

**
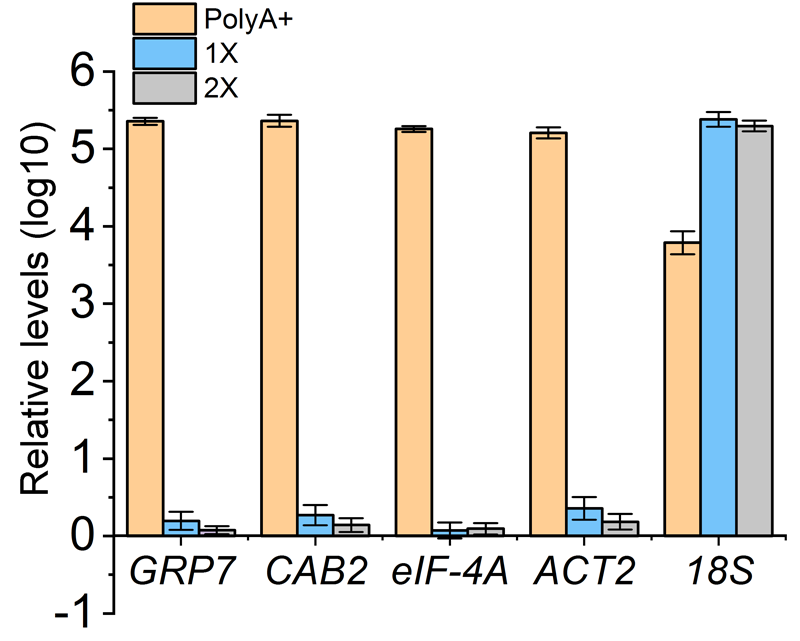
**

**Supplemental Figure S2. Levels of highly abundant polyadenylated transcripts in polyA- fractions.** Quantitative PCR shows levels of highly abundant polyA+ transcripts in two different polyA- RNA fractions of WT. The polyA- RNA fractions 1X and 2X indicate one and two rounds of polyA selection of total RNA, respectively. The polyA+ and polyA- RNA were then primed with oligo(dT)_21_ or random decamer (to amplify 18S) prior to cDNA synthesis. Histograms are means + SD from two biological.

**Supplemental Figure S3**


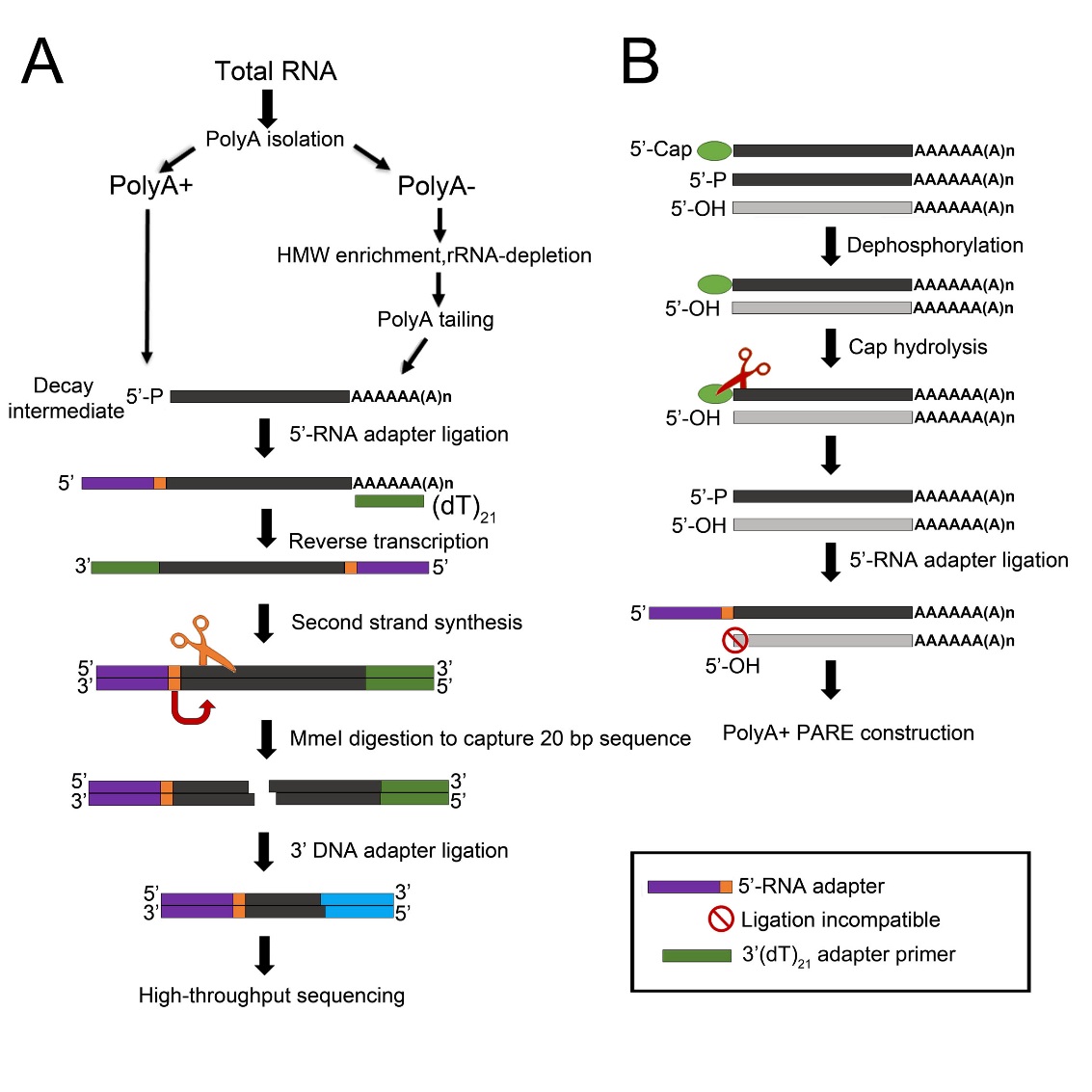


**Supplemental Figure S3. Construction of PolyA+ PARE, PolyA- PARE and Cap-PARE (C-PARE).** (A) For polyA+ and polyA- PARE, total RNA was fractionated to polyA+ and polyA-. PolyA- RNA was enriched for high molecular weight RNA (HMW), rRNA-depleted and then polyA tailed using polyA polymerase. Both polyA+ and polyA- 5’-monophosphate (5’-P) RNA were ligated to the 5’-RNA adapter. RNA was then primed with the 3’(dT)_21_ adapter primer and reverse transcribed. Double stranded DNA (dsDNA) was digested with MmeI which cuts 20 nt downstream of its recognition site*,* and dephosphorylated (not shown). MmeI-treated DNA was ligated to a dsDNA adapter, and PCR amplified prior to deep-sequencing. For more details about PARE library construction see Supplementary Experimental Procedures. (B) C-PARE libraries were constructed as described (1). Briefly, polyA+ RNA was treated with calf-alkaline intestinal phosphatase (CIP, dephosphorylation) prior to cap hydrolysis using tobacco acid pyrophosphatase (TAP). Decapped RNA with 5’-P was ligated to the 5’-RNA adaptor and subsequent steps of library construction are as described in A.

**Supplemental Figure S4**


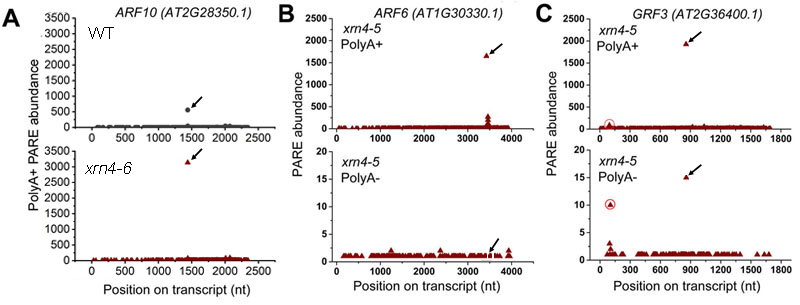


**Supplemental Figure S4. Different accumulation patterns of select 3’ fragments of miRNA-guided cleavage in *xrn4* mutants in polyA+ and polyA- PARE.** D-plots are presented for different miRNA targets in *xrn4* mutants. (A) PolyA+ PARE shows accumulation of 3’ fragment for miRNA target *ARF10* in *xrn4-6* (closed diamonds). (B) Absence of *ARF6* (miR167 target) cleavage product in polyA- as compared to polyA+ in *xrn4-5*. (C) Overaccumulation of 3’ fragment of *GRF3* (miR396 target) in both polyA+ and polyA- in *xrn4-5.* The *xrn4*/WT fold changes at cleavage site in B and C are indicated in Supplemental Table S3. Black arrow, miRNA-guided cleavage site; Red open circle, corresponds to cap site.

**Supplemental Figure S5**


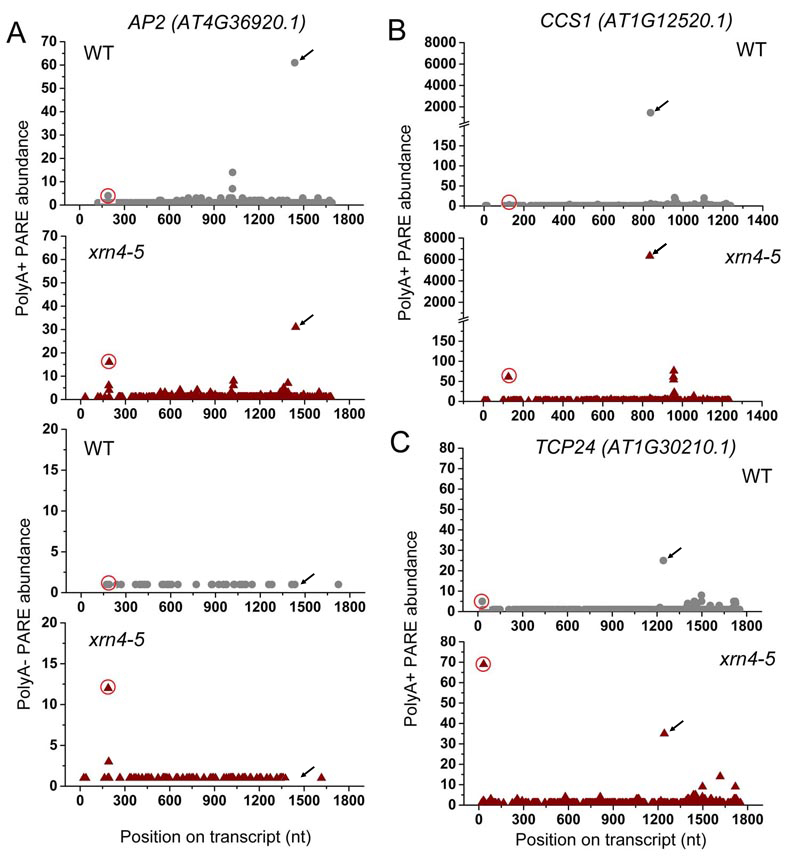


**Supplemental Figure S5. Decapped products of miRNA-targets, *AP2, CCS1* and *TCP24* overaccumulate in *xrn4*.** PARE D-plots show accumulation of decapped and 3’ fragments for miRNA targets in WT (closed circle) and *xrn4-5* (closed triangles) (A) miR172 target *AP2* in polyA+ (top) and polyA- (bottom) PARE. (B) miR398 target *CCS1* in polyA+ PARE and (C) miR319 target *TCP24* in polyA+ PARE. Red open circle, corresponds to the cap site; Black arrow, miRNA-guided cleavage site; Break in y-axis (B) is from 200 and 500 TP20M.

**Supplemental Figure S6**

**
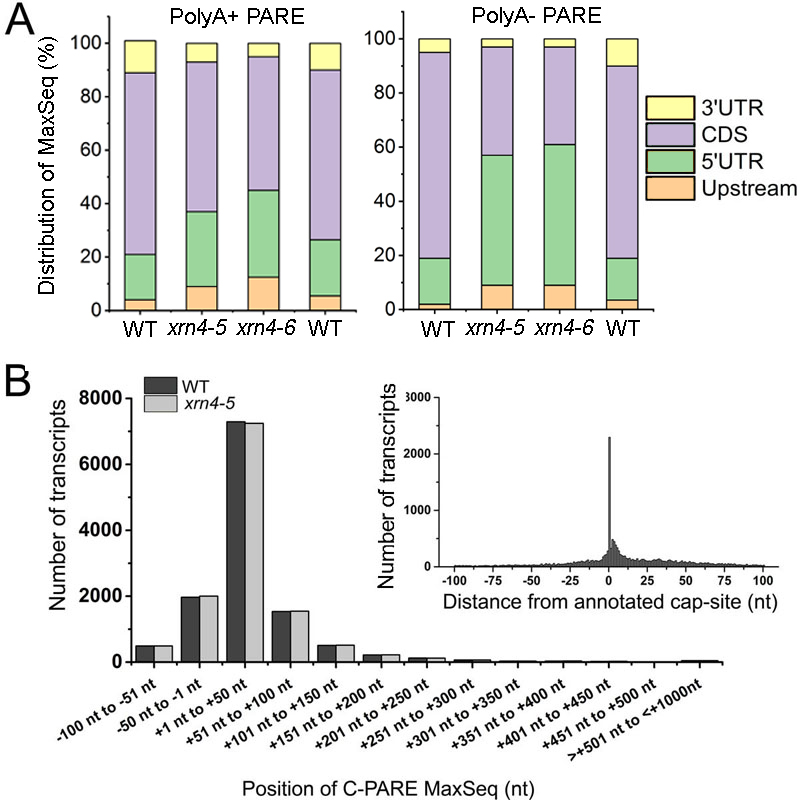
**

**Supplemental Figure S6. Distribution of principal PARE and C-PARE sequences in Arabidopsis transcripts.** (A) Histograms show distribution of MaxSeqs in polyA+ and polyA- PARE libraries. The locations of all MaxSeqs mapping to the indicated regions are presented as a percentage for the different genotypes. Upstream, the 100 nt upstream of and including the annotated transcription start-site (TSS)/cap site; CDS, coding sequence; UTR, untranslated region. (B) Number of transcripts with C-PARE MaxSeqs (y-axis) plotted as a function of position on the transcript (x-axis). Positions are divided into 50 nt intervals except for last interval (+501 to +1000 nt). +1 denotes the TSS/annotated cap site. Inset, distance of C-PARE MaxSeq position from the annotated cap site. Number of transcripts with the same C-PARE MaxSeq in both WT and *xrn4-5* (y-axis); position relative to annotated cap site (x-axis). For description of C-PARE, see Supplemental Figure S3B and Supplementary Experimental Procedures.

**Supplemental Figure S7**

**
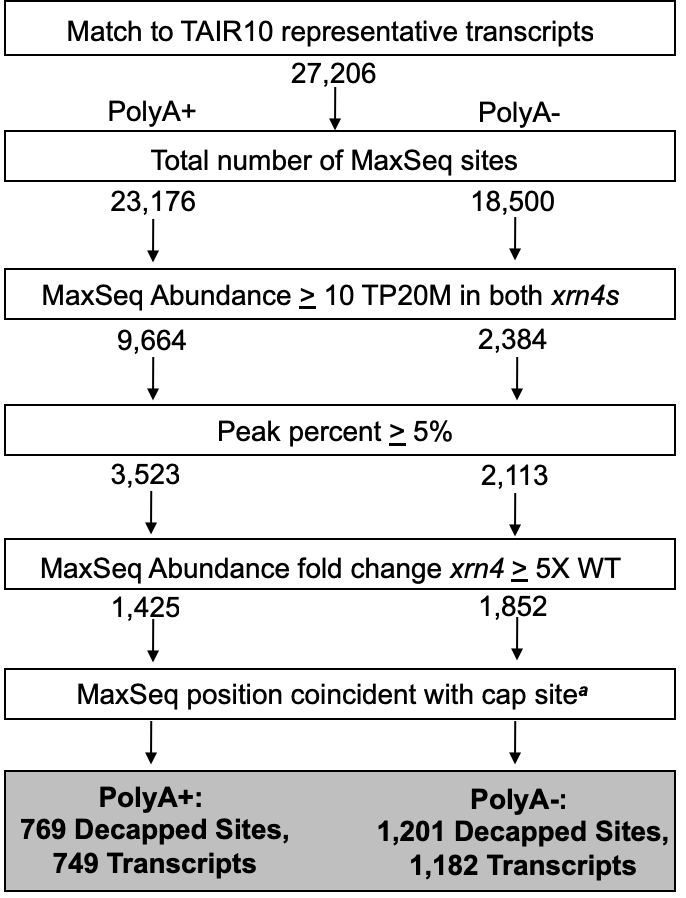
**

**Supplemental Figure S7. Computational pipeline to identify decapped XRN4 substrates from polyA+ and polyA- PARE.** The final outputs of the pipeline highlighted in gray are transcripts classified as decapped XRN4 substrates. A decapped site is a PARE MaxSeq position that coincides with a cap site. The data were filtered using criteria as shown and explained in Supplementary Experimental Procedures. *^a^* cap sites were identified from C-PARE libraries are described in the legend to Supplemental Figure S6 and in the Supplementary Experimental Procedures.

**Supplemental Figure S8**

**
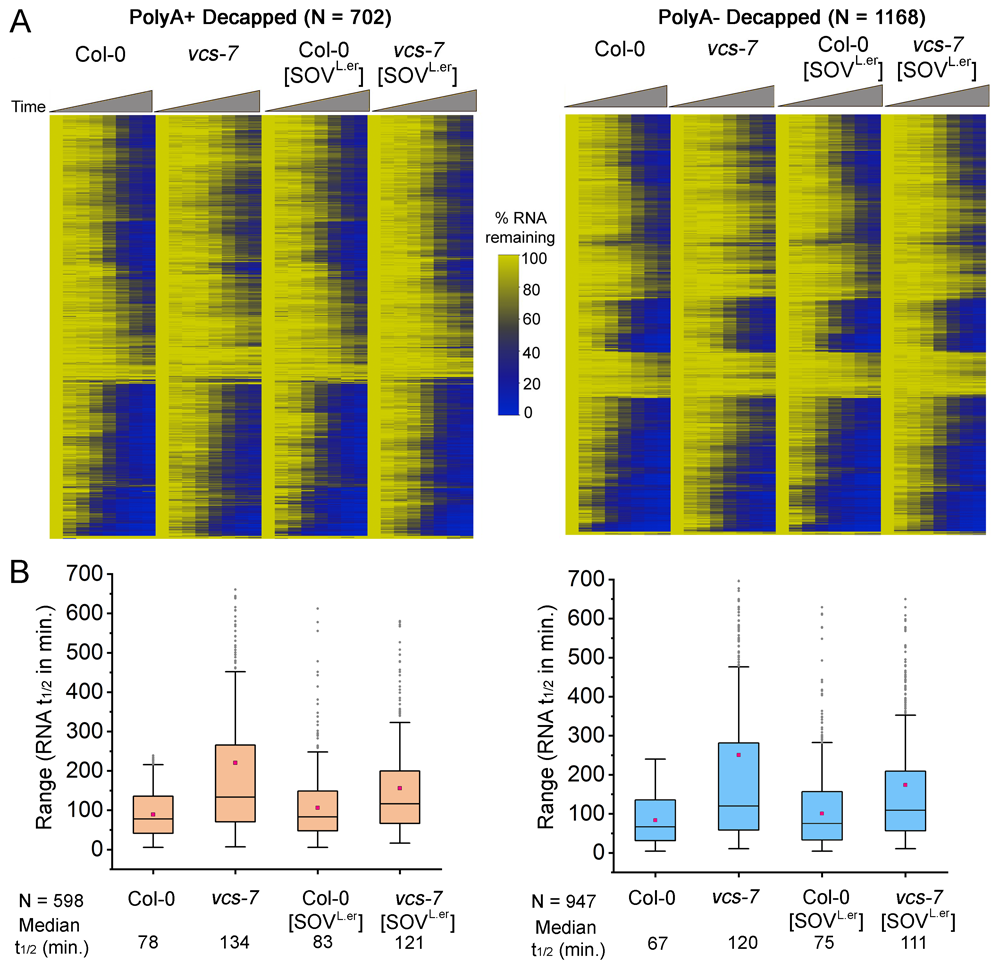
**

**Supplemental Figure S8. RNA decay rates of decapped XRN4 substrates.** (A) The heat maps of RNA decay rates (2) for all polyA+ and polyA- decapped XRN4 substrates in Col-0 ecotype, *vcs-7* (*varicose* mutant in Col-0), Col-0 [SOV^L.er^] (transgenic Col-0 with Landsberg *erecta* copy of SOV, SOV^L.er^) and *vcs-7* [SOV^L.er^] (*vcs-7* carrying SOV^L.er^) are presented. Time, 0, 7.5, 15, 30, 60, 120, 240 and 480 mins after transcription shutoff. RNA abundance at 0 min is set to 100%. (B) Majority of XRN4 substrates show longer RNA *t_1/2_* in the *vcs* mutants. In Col-0, RNA t_1/2_ values were <240 mins for 85% of polyA+ (598 out of 702 transcripts) and 81% of polyA- (947 out of 1,167 transcripts) XRN4 substrates. For these transcripts, box plots show the range of RNA t_1/2_ values in Col-0 and comparative ranges in other genotypes. Legend to the box plots are as per the description in Figure 1C. For (A) and (B) decay rates extracted from (2) are presented in Supplemental Dataset S2.

**Supplemental Figure S9**


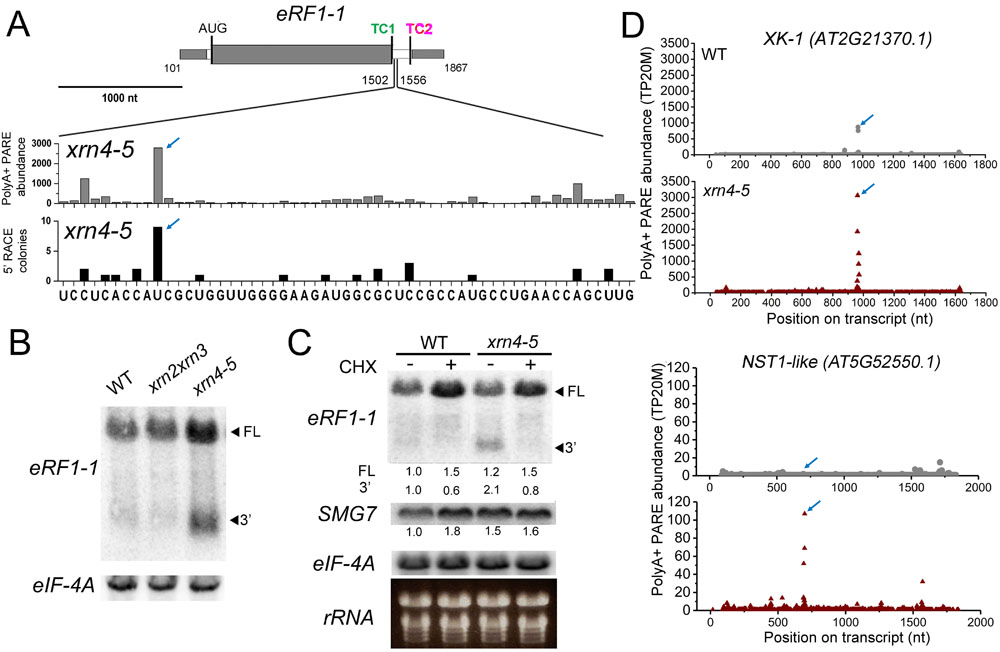


**Supplemental Figure S9. 3’ fragments of some NMD-sensitive transcripts overaccumulate in *xrn4*.** (A) Mapping the position of the 3’ fragment (3’) of *eRF1-1*. Structure of *eRF1-1* mRNA as presented in Figure 4A. A 54 nt region (positions 1402 to 1456 nt) starting 22 nt downstream of the stop codon is expanded to show PARE abundances (gray bars, Figure 4A) and 5’ RACE analysis (black bars) in *xrn4-5*. Red arrow, MaxSeq as shown in Figure 4A. (B) Accumulation of *eRF1-1* 3’ fragment in the inflorescence of WT, *xrn2xrn3* and *xrn4-5*. (C) Effect of translation/NMD inhibitor cyclohexamide (CHX) on *eRF1-1* RNA levels. WT and *xrn4-5* seedlings were treated with either DMSO (solvent) or 10 mg/L CHX for 3h. Total RNA northern blots are shown for B and C and are representative of two or more biological replicates. 3’ probe of *eRF1-1* was as per legend to Figure 4A. Values (in C) indicate fold-changes normalized to those of *eIF-4A* mRNA and WT abundance set to 1. (D) D-plots of other *upf1*-elevated RNAs (*XK-1* above; *NST1-like* below) overaccumulating 3’ fragment in *xrn4.* Blue arrow, MaxSeq position in *xrn4* and its corresponding position in WT.

**Supplemental Figure S10**


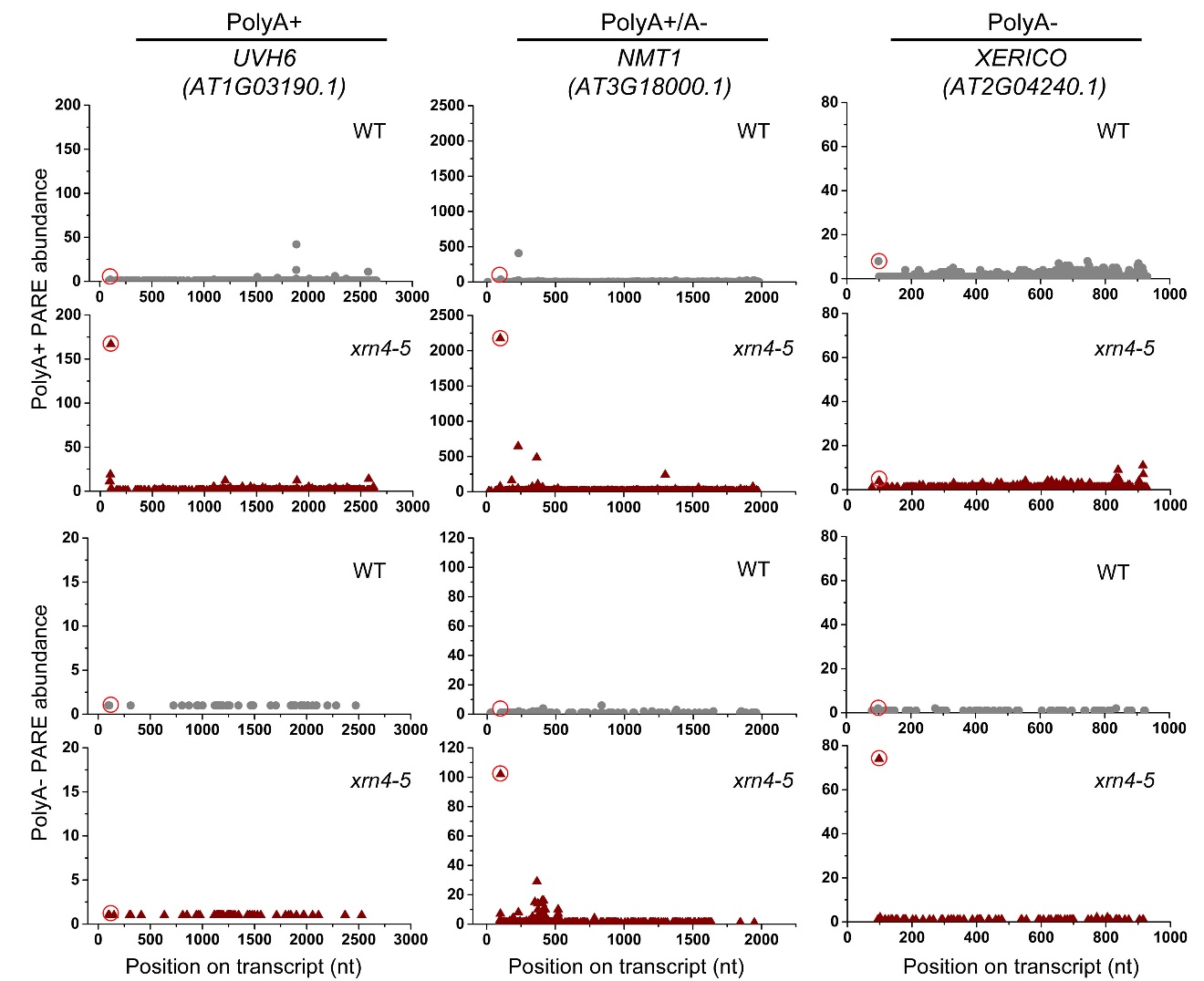


**Supplemental Figure S10. Examples of decapped XRN4 substrates in polyA+ and polyA- PARE.** PARE D-plots show profiles of decapped intermediates for transcripts accumulating as polyA+ (UV-responsive, *UVH6)* in left panel*,* polyA- (osmotic-stress responsive, *XERICO)* in right panel and in both polyA+ and polyA- (CPuORF containing *NMT1)* in center panel. Scale of y-axis in polyA+ and polyA- PARE are not comparable. Red open circle, corresponds to cap site.

**Supplemental Figure S11**


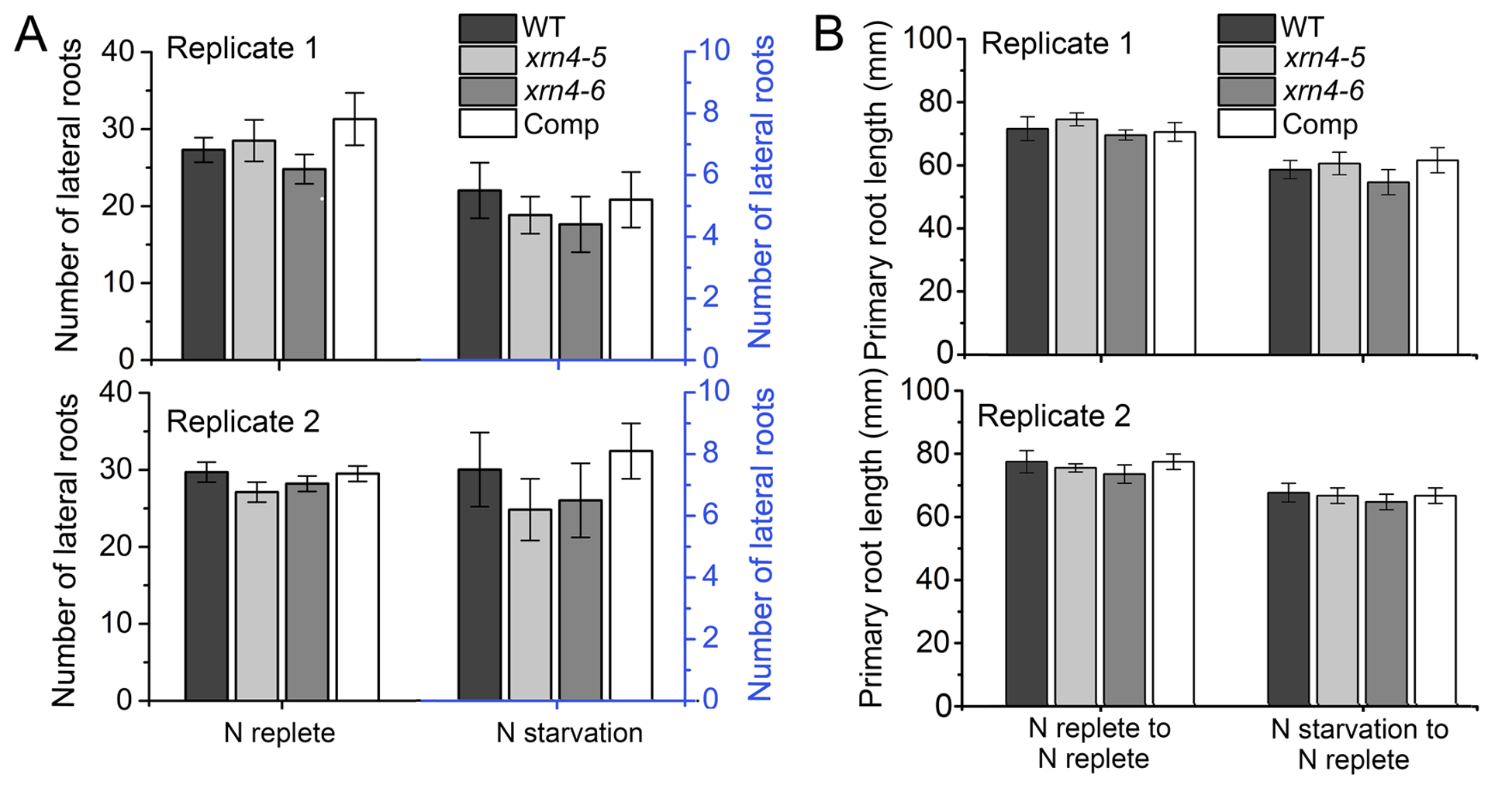


**Supplemental Figure S11. Root parameters without significant change in *xrn4* mutants.** (A) Number of LR of seedlings grown either under control conditions (N-replete medium for 14 d) or N starvation conditions (N-starvation medium for 7 d). Scales on the left and right y-axis are different. (B) Primary root elongation of seedlings grown under control conditions (N-replete medium throughout the experiment) and N resupply conditions (N-starvation medium for 7 d followed by N-replete medium for 7 d). For A and B, histograms are means + SEM of 10 (biological replicate 1) and 15 (biological replicate 2) seedlings per genotype. Experiments were performed two additional times with similar results. None of the mutants differed from WT (*P>0.5*).

**Supplemental Figure S12**


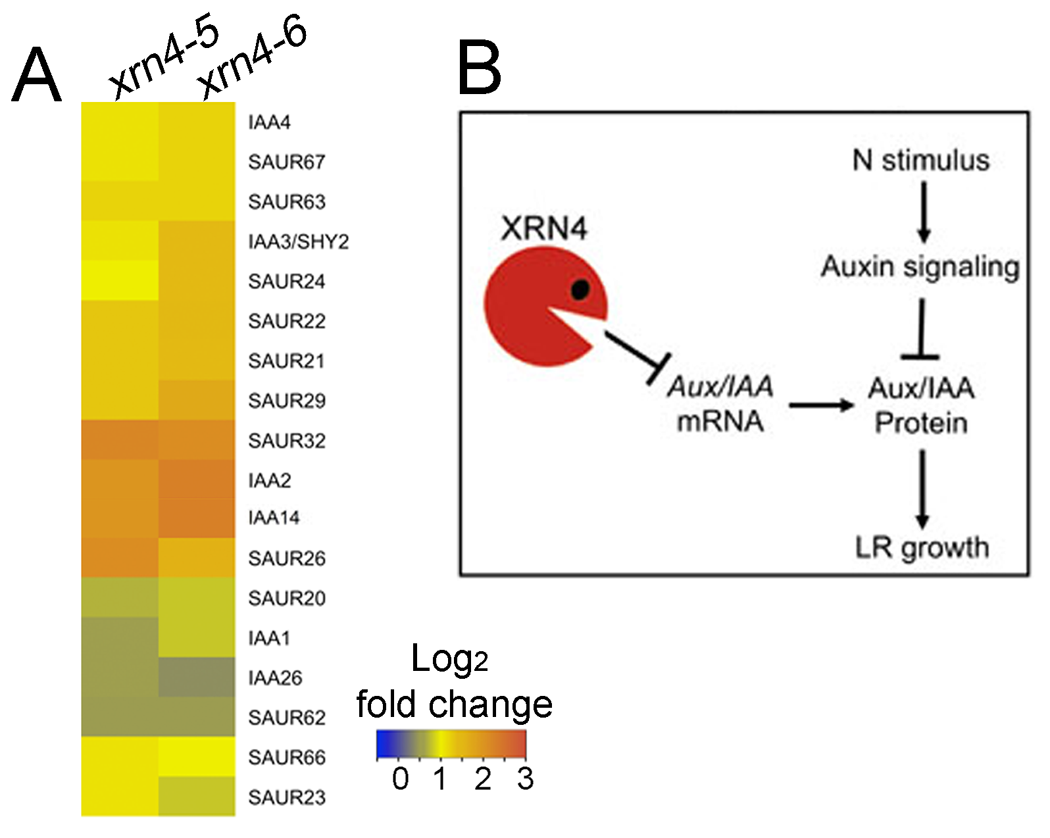


**Supplemental Figure S12. Decapped XRN4 substrates include auxin-responsive transcripts**. (A) The heat map of *SAUR* and *AUX/IAA* transcripts showing fold changes in *xrn4* mutants from polyA- RNA-seq (Supplemental Dataset S1). (B) Potential role of XRN4 in N-responsive LR growth.

**Supplemental Figure S13**

**
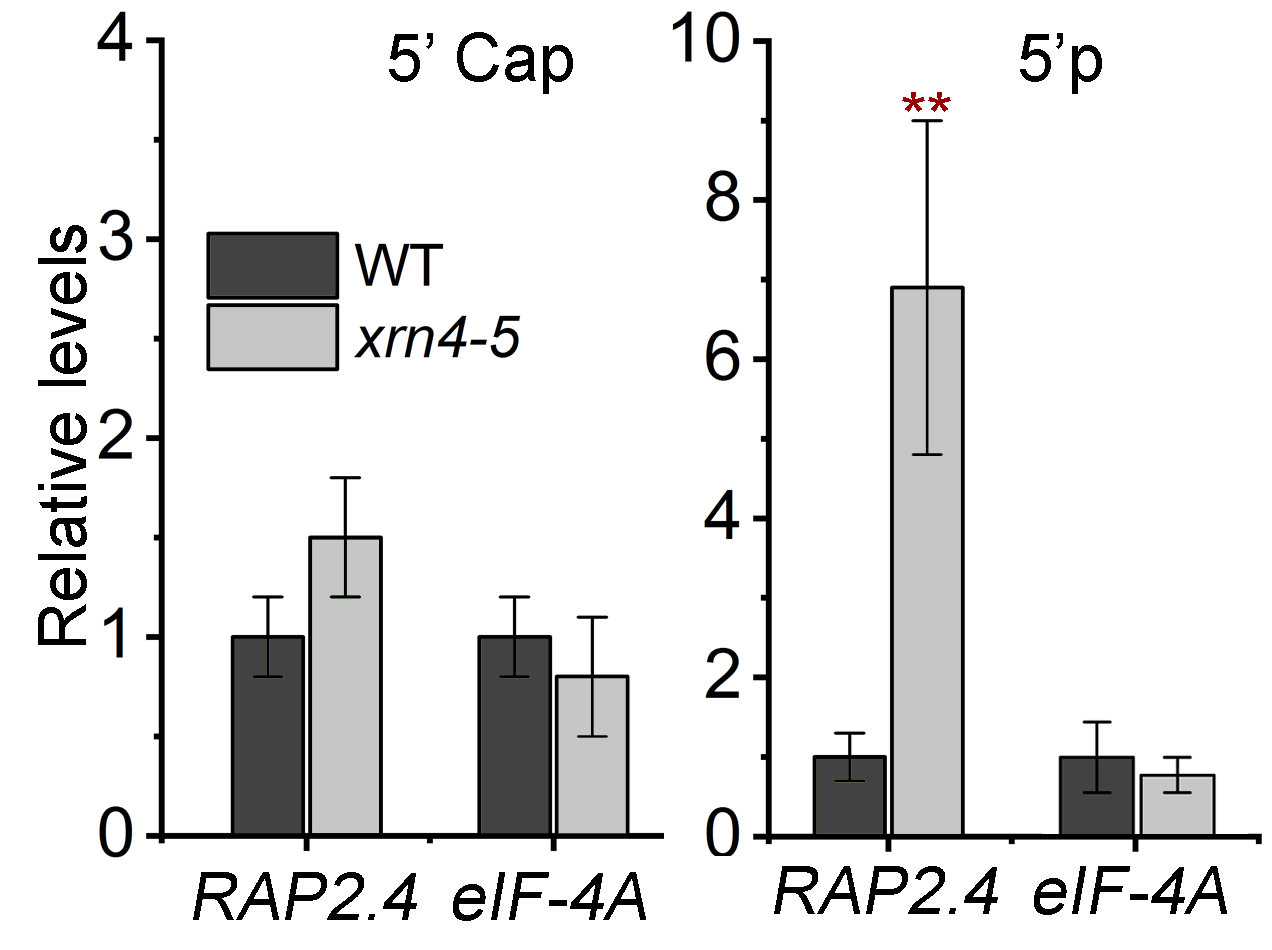
**

**Supplemental Figure S13. Levels of capped and decapped control RNAs.** Quantitative splinted-ligation RT-PCR of total RNA shows decapped (5’ P) and capped (5’ Cap, CIP + TAP) RNA levels for XRN4 substrate *RAP2.4* and non-substrate *eIF-4A* in WT and *xrn4-5* seedlings. No significant difference (*P>0.1*) in 5’ Cap levels of these RNAs between WT and *xrn4-5* was observed. As expected for the XRN4 substrate *RAP2.4*, the decapped transcript was highly elevated in *xrn4-5*. Histograms are means + SD of three biological replicates; **, *P<0.01*.

**Supplementary Tables**

**Table S1. Number of transcripts differentially accumulating in *xrn4* polyA+ and polyA- RNA-seq**

|  |  | ***^1^* Differential accumulation** | |  |
| --- | --- | --- | --- | --- |
|  |  | **Increase** | **Decrease** |  |
|  |  |  |  |  |
| **PolyA+** | ***xrn4-5*** | 140 | 13 |  |
|  | ***xrn4-6*** | 110 | 19 |  |
|  | **Overlap** | 68 | 8 |  |
|  |  |  |  |  |
| **PolyA-** | ***xrn4-5*** | 549 | 8 |  |
|  | ***xrn4-6*** | 648 | 10 |  |
|  | **Overlap** | 459 | 6 |  |
| ***^1^*** Transcripts with RPKM > 5 in WT or *xrn4* mutant with >2-fold (log2 > +1) differential accumulation (FDR adjusted *P<0.05*)  Gray, transcripts represented in Figure 1A | | | | |

**Table S2. Median oligoA lengths and major 5’ ends of deadenylated RNAs**

|  |  |  |  | ***^1^*Major 5’ ends** | |  |
| --- | --- | --- | --- | --- | --- | --- |
| **AGI Number** | **Median oligoA Length (nt)** | ***^2^* Representative Sequence**  **(5’ to 3’)** | **cRT-PCR** | **5’ RLM-RACE** | ***^3^* C-PARE MaxSeq** | ***^4^*Decapped XRN4 substrate** |
| ***xrn4-5*** |  |  |  |  |  |  |
| *AT1G78080*  *(RAP2.4)* | 14 | **AAAAAAAAAAAAAAAAAUUAUCGGUGAGGUUGAG** | -4, 5/19; **1, 14/19** | 27, 3/12; **1, 9/12** | 1 | D |
| *AT3G23030*  *(IAA2)* | 11 | **AAAAAAAAAAUCAACCAGCUCACC** | **1, 8/8** | **1, 9/11**; -1, 2/11 | 1 | D |
| *AT4G34760*  *(SAUR50)* | 10 | **AAAAAAAAAAAACCCAUCUCCUUCUC** | -20, 3/10; **4, 6/10** | **4,10/12** | 4 | D |
| *AT3G25717*  *(DVL6)* | 10 | **AAAAAAAAAAUACUUUCUCUUAUCUA** | **1, 6/7** | **1, 8/9** | 1 | D |
| *AT3G16870*  *(GATA17)* | 9 | **AAAAAAAAAAAACCGCCGUCUCUCU** | -6, 6/10; **3, 4/10** | **3, 9/10** | 1 |  |
| *AT5G20885* | 14 | **AAAAAAAAAAAUCUCUCUCUCUCUCU** | **8, 5/7** | 1, 2/8; **8, 6/8** | ND |  |
| *AT1G22190*  *(WIND2)* | 12 | **AAAAAAAAAAAUUAUCGGUGAGGCUGAG** | 27, 5/14; **29, 8/14** | 27, 4/12; **29, 8/12** | 29 | D |
| *AT5G60850*  *(OBP4)* | 15 | **AAAAAAAAAAAAAUGCUCACCACUUCA** | 5, 3/10; **15, 5/10** | 5, 3/10; **15, 7/10** | 15 | D |
| *AT3G18780*  *(ACT2)* | 9 | **AAAAAAAAAGUCUCGUUGUCCUCC** | 43, 3/9; **76, 5/9** | **76, 8/10;** 77, 2/10 | 76 |  |
| *AT4G05320*  *(UBC)* | 12 | **AAAAAAAAAAAAAACAAUUCAGAUUUCA** | **19, 5/7** | **19, 8/8** | 19 |  |
| Median (nt) | **12** |  |  |  |  |  |
|  |  |  |  |  |  |  |
| **WT** |  |  |  |  |  |  |
| *AT1G78080*  *(RAP2.4)* | 13 | **AAAAAAAAAAAAUAUCGGUGAGGUUGAG** | -4, 2/8; **1, 4/8** |  |  |  |
| *AT1G22190*  *(WIND2)* | 12 | **AAAAAAAAAAAAUAUCGGUGAGGCUGAG** | 27, 2/6; **29, 4/6** |  |  |  |
| *AT5G60850*  *(OBP4)* | 13 | **AAAAAAAAAAAUGCUCACCACUUCA** | **15, 3/6** |  |  |  |
| *AT3G18780*  *(ACT2)* | 9 | **AAAAAAAGUCUCGUUGUCCUCC** | 43, 2/8; **76, 4/8** |  |  |  |
| *AT4G05320*  *(UBC)* | 11 | **AAAAAAAAAAAAACAAUUCAGAUUUCA** | **19, 4/7** |  |  |  |
| Median (nt) | **12** |  |  |  |  |  |

***^1^*** Position followed by frequency are indicated for the two most frequent 5’ ends. Position 1 is the annotated 5’ end; Orange indicates the major cloned products common to cRT-PCR of polyA- RNA and 5’RLM-RACE of capped polyA+ RNA (decapped *in vitro*)

***^2^*** Chosen for the orange major 5’ end and secondarily, the closest to the median oligoA length. Sequences 3’ of the last nt matching the 3’UTR are shown.

***^3^*** Position relative to 1 being the annotated 5’ end of transcript. ND, No cap site detected in C-PARE analysis

***^4^*** Decapped XRN4 substrates (D) are presented in Supplemental Dataset S2

**Table S3. Abundances of 3’ cleavage fragments of miRNA targets overaccumulating in *xrn4* mutants in polyA+ and polyA- PARE**

|  |  |  | PolyA+ PARE | | | | | | PolyA- PARE | | | | | |
| --- | --- | --- | --- | --- | --- | --- | --- | --- | --- | --- | --- | --- | --- | --- |
| ***^1^***AGI Number | miRNA | Cleavage Position | WT | *xrn4-5* | FC | WT | *xrn4-6* | FC | WT | *xrn4-5* | FC | WT | *xrn4-6* | FC |
| AT1G08830.1 | mir398A | 228 | 198 | 304 | 0.6 | 288 | 520 | 0.9 | 0 | 1 | 0.0 | 1 | 5 | 2.3 |
| AT1G10120.1 | mir396 | 1417 | 98 | 317 | 1.7 | 224 | 589 | 1.4 | 0 | 1 | 0.0 | 1 | 0 | 0.0 |
| AT1G27340.1 | mir394 | 1483 | 27 | 43 | 0.7 | 52 | 75 | 0.5 | 0 | 0 | 0.0 | 0 | 0 | 0.0 |
| AT1G27360.1 | mir156G | 1363 | 295 | 865 | 1.6 | 658 | 992 | 0.6 | 1 | 1 | 0.0 | 0 | 0 | 0.0 |
| AT5G10180.1 | mir395E | 236 | 13 | 25 | 0.9 | 15 | 77 | 2.4 | 1 | 0 | 0.0 | 0 | 0 | 0.0 |
| AT1G69170.1 | mir156G | 1407 | 52 | 327 | 2.7 | 193 | 468 | 1.3 | 1 | 1 | 0.0 | 0 | 2 | 1.0 |
| AT5G07680.1 | mir164B | 954 | 4 | 28 | 2.8 | 16 | 27 | 0.8 | 0 | 0 | 0.0 | 0 | 0 | 0.0 |
| AT2G36400.1 | mir396A | 857 | 174 | 1939 | 3.5 | 1106 | 3132 | 1.5 | 1 | 15 | 3.9 | 1 | 33 | 5.0 |
| AT3G60250.1 | mir397B | 343 | 162 | 575 | 1.8 | 282 | 769 | 1.4 | 0 | 11 | 3.5 | 0 | 16 | 4.0 |
| AT1G30490.1 | mir165 | 906 | 328 | 2237 | 2.8 | 1084 | 2877 | 1.4 | 1 | 3 | 0.6 | 2 | 3 | 1.6 |
| AT4G24150.1 | mir396 | 948 | 17 | 116 | 2.8 | 56 | 185 | 1.7 | 0 | 0 | 0.0 | 1 | 1 | 0.0 |
| AT2G28350.1 | mir160C | 1440 | 603 | 2109 | 1.8 | 801 | 3206 | 2.0 | 2 | 21 | 4.4 | 1 | 19 | 3.2 |
| AT5G60690.1 | mir165A | 1373 | 70 | 561 | 3.0 | 207 | 535 | 1.4 | 0 | 1 | 0.0 | 1 | 1 | 0.0 |
| AT3G60630.1 | mir171B | 1153 | 636 | 3417 | 2.4 | 1192 | 4182 | 1.8 | 3 | 8 | 3.0 | 1 | 13 | 2.1 |
| AT3G57920.1 | mir156G | 955 | 24 | 45 | 0.9 | 72 | 131 | 0.9 | 1 | 1 | 0.0 | 0 | 0 | 0.0 |
| AT4G30080.1 | mir160C | 1619 | 589 | 4725 | 3.0 | 1513 | 4687 | 1.6 | 2 | 22 | 2.9 | 3 | 26 | 3.7 |
| AT1G17590.1 | mir169F | 1343 | 89 | 706 | 3.0 | 217 | 458 | 1.1 | 0 | 0 | 0.0 | 0 | 0 | 0.0 |
| AT4G00150.1 | mir170A | 967 | 54 | 180 | 1.7 | 194 | 307 | 0.7 | 0 | 1 | 0.0 | 0 | 3 | 1.6 |
| AT4G03190.1 | mir393 | 1698 | 8 | 35 | 2.1 | 55 | 107 | 1.0 | 0 | 0 | 0.0 | 1 | 2 | 1.0 |
| AT1G66700.1 | mir163 | 458 | 89 | 252 | 1.5 | 70 | 750 | 3.4 | 0 | 0 | 0.0 | 0 | 0 | 0.0 |
| AT5G37020.1 | mir167D | 2481 | 26 | 304 | 3.5 | 81 | 204 | 1.3 | 0 | 1 | 0.0 | 1 | 0 | 0.0 |
| AT4G32880.1 | mir165 | 1045 | 16 | 65 | 2.0 | 50 | 145 | 1.5 | 0 | 1 | 0.0 | 0 | 0 | 0.0 |
| AT4G37740.1 | mir396 | 1021 | 446 | 1061 | 1.3 | 818 | 2004 | 1.3 | 1 | 2 | 1.0 | 1 | 3 | 1.6 |
| AT2G34010.1 | mir159B | 549 | 30 | 146 | 2.3 | 38 | 192 | 2.3 | 0 | 0 | 0.0 | 0 | 0 | 0.0 |
| AT4G00150.1 | mir171B | 964 | 31 | 379 | 3.6 | 86 | 452 | 2.4 | 0 | 0 | 0.0 | 0 | 4 | 2.0 |
| AT5G61430.1 | mir164B | 951 | 10 | 223 | 4.5 | 50 | 109 | 1.1 | 0 | 0 | 0.0 | 0 | 0 | 0.0 |
| AT2G02850.1 | mir408 | 207 | 239 | 1655 | 2.8 | 267 | 1007 | 1.9 | 0 | 1 | 0.0 | 1 | 1 | 0.0 |
| AT2G34710.1 | mir165 | 982 | 9 | 209 | 4.5 | 20 | 185 | 3.2 | 0 | 0 | 0.0 | 0 | 0 | 0.0 |
| AT1G12520.1 | mir398 | 836 | 766 | 6352 | 2.1 | 1476 | 4441 | 2.5 | 2 | 1 | 0.0 | 1 | 3 | 0.6 |
| AT1G12820.1 | mir393 | 1995 | 165 | 18 | -3.1 | 155 | 80 | -1.0 | 6 | 17 | 1.5 | 6 | 17 | 1.5 |
| AT1G77850.1 | mir160C | 1520 | 530 | 67 | -2.0 | 259 | 235 | -1.2 | 2 | 10 | 3.3 | 1 | 13 | 2.7 |
| AT2G45160.1 | mir171A | 1114 | 4440 | 5746 | 0.4 | 8204 | 10067 | 0.3 | 8 | 36 | 2.8 | 5 | 70 | 3.1 |

***^1^*** Highlighted transcript showed an abundance at miRNA-target cleavage site > 10 TP20M and log2 fold-change (FC) > 0.6 (i.e. 1.5) in both *xrn4-5* and *xrn4-6*: orange, in polyA+; blue, in polyA-; gray, in both polyA+ and polyA-.

**Table S4. GeneSect analysis and datasets used in the study**

|  |  |  | |  |  | |  | | | **Overlap with decapped** | | | | | | | |  |  |  |
| --- | --- | --- | --- | --- | --- | --- | --- | --- | --- | --- | --- | --- | --- | --- | --- | --- | --- | --- | --- | --- |
| ***^1^* Decapped** | | | | | | | | | | **GeneSect** | | | | | | | |  |  |  |
|  |  |  |  |  |  |  |  |  |  | **PolyA+ (749)** | | | **PolyA- (1182)** | | | | |  |  |  |
| Dataset | Ref. | Data type | | Filters used in the published study | No. of transcripts (protein coding) | | ***^2^*** N | | | **Overlap** | ***^3^* z score** | | **Overlap** | | ***^3^* z score** | | |  |  |  |
| NEC | [1] |  | | | 1,476 | | 1,190 | | | **53** | **3.9** | | **145** | | **6.5** | | |  |  |  |
| Heat repressed | [2] | RNA-seq | | Fold Change (FC): >2 38C/22C in WT; FDR P<0.05 | 3,005 | | 1,780 | | | **98** | **1.4** | | **203** | | **4.3** | | |  |  |  |
| Heat induced | [2] | RNA-seq | | FC: >2 38C/22C in WT; FDR P<0.05 | 123 | | 74 | | | **7** | **1.8** | | **11** | | **1.9** | | |  |  |  |
| *upf1-5* elevated | [3] | Microarray | | FC: > 1.5 *upf1* /WT; FDR P<0.05 | 1,138 | | 390 | | | **39** | **5.7** | | **37** | | **0.5** | | |  |  |  |
| *upf3-1* elevated | [3] | Microarray | | FC: > 1.5 *upf3* /WT; FDR P<0.05 | 1,220 | | 438 | | | **68** | **8.1** | | **44** | | **1.1** | | |  |  |  |
| N induced | [4] | Microarray | | FC: >1.5 KNO3/KCl in WT shoots | 486 | | 399 | | | **20** | **0.2** | | **62** | | **7.7** | | |  |  |  |
| NO3 responsive | [5] | RNA-seq | | FC: >2 KNO3/KCl in WT; FDR P<0.05 | 505 | | 460 | | | **15** | **-0.8** | | **51** | | **5.6** | | |  |  |  |
| N resupply | [6] | Microarray | | Transcription factors induced or repressed within 30 min. of N resupply; FC: > 3; FDR P<0.05 | 93 | | 57 | | | **4** | **0.1** | | **19** | | **6.1** | | |  |  |  |
| CPuORF transcripts | [7,8] |  | | | 80 | | 47 | | | **9** | **2.1** | | **14** | | **5.8** | | |  |  |  |
| ***^4^* 3’ fragment** | | | | | | | | | **Overlap with 3’ fragments** | | | |  | | |  | | |  |  |
|  |  |  |  |  |  |  |  |  | **PolyA+ (568)** | | | |  | | |  | | |  |  |
| Dataset | Ref. | | Data type | Filters used in the published study | | No. of transcripts (protein coding) | | ***^2^*** N | **GeneSect** | | | |  | **P values** | | |  |  |  |  |
|  |  | |  |  | |  | |  | **Overlap** | | | ***^3^* z score** |  | ***<0.001*** | | |  |  |  |  |
| *upf1-5* elevated | [3] | | microarray | FC: > 1.5 *upf1/* WT; FDR P<0.05 | | 1,138 | | 390 | **37** | | | **4.8** |  | ***<0.01*** | | |  |  |  |  |
| *upf3-1* elevated | [3] | | microarray | FC: > 1.5 *upf3/* WT; FDR P<0.05 | | 1,220 | | 438 | **63** | | | **8.3** |  | ***<0.05*** | | |  |  |  |  |
| CPuORF transcripts | [7,8] | |  | | | 80 | | 47 | **9** | | | **6.1** |  | ***No change*** | | |  |  |  |  |
| ***^1^*** Decapped XRN4 substrates, see Supplemental Figure S6 | | | | | | | | | | | | | | | | | | | | |
| ***^2^*** N, Number of transcripts in the dataset with an identified cap site used in the overlap analysis  ***^3^*** z scores in red font indicate significant overlap between XRN4 substrates and gene lists  ***^4^*** 3’ fragments: MaxSeq at same site in both *xrn4* mutants, abundance > 20 TP20M and fold change > 2 WT (Supplementary experimental procedures). | | | | | | | | | | | | | | | | | | | | |

**Table S5. Gene Ontology (GO) categories for biological processes impacted in both *xrn4* mutants**

| PARE: Decapped XRN4 Substrates | | | |
| --- | --- | --- | --- |
|  | Gene Ontology (GO) Category | Fold Enrichment | P-value |
| **PolyA+** | GO:0009768~photosynthesis | 8.5 | 0.0004 |
|  | GO:0009637~response to blue light | 3.0 | 0.0050 |
|  | GO:0018298~protein-chromophore linkage | 2.5 | 0.0030 |
|  | GO:0000398~mRNA splicing | 2.2 | 0.0090 |
|  | GO:0010200~response to chitin | 2.1 | 0.0000 |
|  | GO:0006457~protein folding | 5.1 | 0.0003 |
|  | GO:0006397~mRNA processing | 4.2 | 0.0070 |
|  | GO:0042254~ribosome biogenesis | 2.4 | 0.0001 |
|  | GO:0012501~programmed cell death | 6.3 | 0.0110 |
| **PolyA-** | GO:0002181~cytoplasmic translation | 5.2 | 0.0000 |
|  | GO:0006833~water transport | 5.1 | 0.0000 |
|  | GO:0010114~response to red light | 4.0 | 0.0001 |
|  | GO:0015979~photosynthesis | 3.7 | 0.0000 |
|  | GO:0009733~response to auxin | 2.2 | 0.0001 |
|  | GO:0010200~response to chitin | 3.2 | 0.0000 |
|  | GO:0042254~ribosome biogenesis | 3.0 | 0.0000 |
|  | GO:0009735~response to cytokinin | 3.1 | 0.0000 |
|  | GO:0009416~response to light stimulus | 2.9 | 0.0000 |
|  | GO:0009611~response to wounding | 2.7 | 0.0000 |
|  | GO:0046686~response to heavy metal ion | 2.6 | 0.0000 |
|  | GO:0009409~response to cold | 2.5 | 0.0000 |
|  | GO:0009651~response to salt stress | 2.2 | 0.0000 |
|  | GO:0006457~protein folding | 2.1 | 0.0000 |
|  | GO:0009753~response to jasmonic acid | 2.3 | 0.0006 |
|  | GO:0006334~nucleosome assembly | 3.4 | 0.0009 |
|  | GO:0018298~protein-chromophore linkage | 3.7 | 0.0003 |
|  | GO:0009631~cold acclimation | 2.9 | 0.0010 |
|  | GO:0006970~response to osmotic stress | 2.1 | 0.0080 |
|  | GO:0034605~cellular response to heat | 4.0 | 0.0090 |
|  | GO:0009744~response to sucrose | 3.2 | 0.0060 |
|  | GO:0042546~cell wall biogenesis | 3.1 | 0.0060 |
|  | GO:0000103~sulfate assimilation | 3.3 | 0.0030 |
|  | GO:0009637~response to blue light | 2.8 | 0.0090 |
|  | GO:0080167~response to karrikin | 2.3 | 0.0090 |

| RNA-seq: *xrn4*-elevated transcripts | | | |
| --- | --- | --- | --- |
|  | Gene Ontology (GO) Category | Fold Enrichment | P-value |
| **PolyA+** | GO:0010033~response to organic substance | 5.2 | 0.0000 |
|  | GO:0009725~response to hormone stimulus | 5.2 | 0.0000 |
|  | GO:0010200~response to chitin | 15.7 | 0.0000 |
|  | GO:0009743~response to carbohydrate stimulus | 10.7 | 0.0000 |
|  | GO:0009415~response to water | 9.3 | 0.0000 |
|  | GO:0006869~lipid transport | 13.2 | 0.0000 |
|  | GO:0009737~response to abscisic acid stimulus | 6.1 | 0.0000 |
|  | GO:0009793~embryo development ending in seed dormancy | 5.7 | 0.0000 |
|  | GO:0009628~response to abiotic stimulus | 2.8 | 0.0000 |
|  | GO:0009723~response to ethylene stimulus | 9 | 0.0001 |
|  | GO:0009612~response to mechanical stimulus | 23 | 0.0002 |
|  | GO:0051252~regulation of RNA metabolic process | 3.6 | 0.0002 |
|  | GO:0022414~reproductive process | 3.2 | 0.0005 |
|  | GO:0009733~response to auxin stimulus | 5.1 | 0.0011 |
|  | GO:0009755~hormone-mediated signaling pathway | 5.7 | 0.0012 |
|  | GO:0051171~regulation of nitrogen metabolism | 2.4 | 0.0012 |
|  | GO:0080003~thalianol metabolic process | 16 | 0.0045 |
|  | GO:0080090~regulation of primary metabolic process | 2.2 | 0.0047 |
|  | GO:0019222~regulation of metabolic process | 2 | 0.0100 |
|  | GO:0071310~cellular response to organic substance | 3.9 | 0.0100 |
|  | GO:0032502~developmental process | 2 | 0.0100 |
| **PolyA-** | GO:0009719~response to endogenous stimulus | 3.3 | 0.0000 |
|  | GO:0009725~response to hormone stimulus | 3.5 | 0.0000 |
|  | GO:0045449~regulation of transcription | 2.7 | 0.0000 |
|  | GO:0051171~regulation of nitrogen metabolism | 2.5 | 0.0000 |
|  | GO:0080090~regulation of primary metabolic process | 6.8 | 0.0000 |
|  | GO:0009733~response to auxin stimulus | 12.1 | 0.0000 |
|  | GO:0051252~regulation of RNA metabolic process | 7.4 | 0.0000 |
|  | GO:0009723~response to ethylene stimulus | 4.7 | 0.0000 |
|  | GO:0010200~response to chitin | 4.7 | 0.0000 |
|  | GO:0009409~response to cold | 6.7 | 0.0000 |
|  | GO:0009266~response to temperature stimulus | 4.9 | 0.0001 |
|  | GO:0009414~response to water deprivation | 3.5 | 0.0001 |
|  | GO:0009737~response to abscisic acid stimulus | 2.8 | 0.0002 |
|  | GO:0009415~response to water | 3.5 | 0.0002 |
|  | GO:0009628~response to abiotic stimulus | 1.8 | 0.0003 |
|  | GO:0009743~response to carbohydrate stimulus | 3.2 | 0.0008 |
|  | GO:0009611~response to wounding | 6.9 | 0.0010 |
|  | GO:0006970~response to osmotic stress | 2.1 | 0.0100 |

**SUPPLEMENTARY EXPERIMENTAL PROCEDURES**

*Library construction*

Strand-specific RNA-seq: Total RNA was extracted using the TRI reagent (Thermo Fisher Scientific) as per the manufacturer’s instructions, with the exception of a chloroform extraction step prior to isopropanol precipitation. PolyA+ RNA was fractionated from total RNA (100 μg) using a standard RNA magnetic bead-based oligo(dT) purification (Absolutely mRNA, Agilent Technologies Inc.). The resultant polyA+ RNA was then purified again using the same kit to enrich polyA+ species, whereas the unbound RNA was saved as the deadenylated polyA- fraction. qRT-PCR analysis showed levels of highly abundant polyadenylated transcripts to be extremely low in the polyA- fractions after one or two rounds of oligo(dT) purifications of WT total RNA, validating the efficacy of our fractionation (Supplemental Figure S2). PolyA- RNA (5 μg) was rRNA-depleted using the Ribo-Zero leaf rRNA removal kit v2 (Epicenter, Illumina Inc.) as per the manufacturer’s instructions. Quality and yield of RNA were checked on the bioanalyzer using the plant RNA pico kit. Approximately 50 ng of RNA (polyA+ or polyA-) was used for the construction of strand-specific RNA-seq libraries using the ScriptSeq v2 kit (Epicenter, Illumina Inc.) following the manufacturer’s instructions. Size-distribution and concentration of the libraries were estimated on the bioanalyzer using the High-Sensitivity DNA kit (Agilent Technologies Inc.). RNA-seq libraries were sequenced using the Illumina HiSeq 2500.

PARE libraries: Total RNA (200 μg) was fractionated as for the RNA-seq libraries. PolyA+ PARE libraries were constructed as described previously, with minor modifications (see Supplemental Figure S2A (1, 3, 4)). PolyA+ RNA (1 μg) was ligated to the 5’ PARE RNA adapter using T4 RNA ligase at 37^o^C. As previously described, the 5’ PARE RNA adapter contains the M*meI* restriction enzyme recognition site (3). RNA was then purified again using the mRNA isolation kit. Adapter-ligated RNA was primed with the 3’ PARE (dT)_21_ adapter and reverse transcribed using SuperScript II (Thermo Fisher Scientific) at 42^o^C. The dsDNA amplified from the cDNA using 5’ and 3’ adapter specific primers was then digested with *MmeI* (NEB Inc.) followed by treatment with alkaline phosphatase at 37^o^C. The digested DNA was resolved in a 15% non-denaturing PAGE gel and a band corresponding to 42 nt was excised. This fragment was ligated to TruSeq dsDNA adapter (Illumina Inc.) using T4 DNA ligase (Thermo Fisher Scientific). Adapter-ligated DNA was amplified by 21-cycle PCR using the 5’ adapter primer and 3’ TruSeq barcode primers (Illumina Inc.). The PCR reactions were resolved on a 6% non-denaturing PAGE gel to detect a 126 nt band corresponding to the final PARE library product. Library concentrations were estimated on the bioanalyzer using the High-Sensitivity DNA kit (Agilent Technologies Inc.). For quality control checks, the library product was cloned into the pCR Zero Blunt cloning vector (Thermo Fisher Scientific) and 24 colonies per library were sequenced using a big-dye reaction (MCLab Inc.). PARE libraries were sequenced using the Illumina HiSeq 2500 platform.

For the construction of PolyA- PARE, the polyA-depleted fraction (100 μg) was enriched for high-molecular weight (HMW) RNA using PEG/NaCl twice before desalting with RNeasy clean-up kit (QIAGEN). PolyA- RNA (5 μg) was then rRNA-depleted using Ribo-Zero leaf rRNA removal kit v2 (Epicenter, Illumina Inc.). The final yield was 500 ng of RNA per preparation. Total of 1 μg RNA was then treated with polyA polymerase (Tailing kit, Epicenter, Illumina Inc.) and incubated at 37^o^C for 1 h. The A-tailed RNA was purified using the mRNA isolation kit and ligated to the 5’ PARE RNA adapter. The subsequent steps are as described for the polyA+ PARE construction. Cap PARE (C-PARE) libraries were constructed as described ((1), Supplemental Figure S2B) Briefly, polyA+ RNA (2 μg) was treated with calf intestinal alkaline phosphatase and incubated at 50^o^C for 1h. The dephosphorylated RNA was then treated with tobacco acid pyrophosphatase (TAP) at 37^o^C for 1 h to hydrolyze mRNA caps. The decapped RNA was then ligated to the 5’ PARE RNA adapter as detailed in polyA+ PARE construction. All enzymatic steps during library preparation were followed by sequential clean-up of the reaction using phenol and chloroform, followed by ethanol precipitation at -80^o^C.

*5’ RACE, Splinted-ligation (SL) and Circular RT-PCR analysis*: A modified RNA ligase-mediated (RLM) 5’RACE was performed using the FirstChoice RLM-RACE kit (Thermo Fisher Scientific) as described (5–7). Total RNA (5 µg), polyA+ (100 ng) or polyA- (10 µg) RNA was ligated to 5’-RACE RNA adapter and reverse transcribed using random decamer primer. After an initial round of PCR amplification (25 to 30 cycles) of the cDNA using adapter- and gene-specific outer primers, a second round of PCR amplification was performed on the resulting dsDNA with internal (adapter- and gene-specific) primers for quantitative RT-PCR analysis or for cloning the 5’ ends. To evaluate the 5’ Cap abundance and position, classical 5’ RACE was used where the RNA was sequentially treated with calf-alkaline phosphatase (CIP) and tobacco acid pyrophosphatase (TAP) prior to ligation with the RNA adapter. Splinted-ligation RT-PCR was performed as described previously (2). In brief, uncapped or *in vitro* decapped (CIP-TAP treated) RNA was mixed with 33 μM RNA-adapter (anchor) and 20 μM splint DNA oligonucleotide and sequentially incubated for 5 min from 70^o^C to 60 ^o^C to 42 ^o^C, and finally to 25 ^o^C. Ligation was carried out at 16 ^o^C overnight with T4 DNA ligase. Splint DNA was digested with DNaseI prior to cDNA synthesis. Gene-specific and anchor-specific primers were used to amplify the cDNA. PCR amplified products were gel purified, cloned into the pCR Zero Blunt cloning vector and sequenced.

For circular RT-PCR (cRT-PCR), polyA- RNA (10 µg) was circularized with T4 RNA ligase and 10 mM ATP as described (8), and reverse transcribed using a gene-specific reverse primer. The cDNA was amplified with primers in antisense (5’) and sense (3’) orientations flanking the head-to-tail junction region of the reverse transcribed product. The PCR products were handled as described in the 5’RACE assays.

*Computational analyses*

RNA-seq libraries: All sequences were checked for quality using FastQC v0.11 (<http://www.bioinformatics.babraham.ac.uk/projects/fastqc/)> and FastX toolkit (hannonlab.cshl.edu) was used to trim adapter sequences and remove low quality reads. The remaining reads were mapped to the Arabidopsis TAIR10 reference genome (July 2016; <ftp://ftp.ensemblgenomes.org/pub/plants/release-43/fasta/arabidopsis_thaliana>) using TopHat v2.1 (9, 10) with following parameters: *allowing one mismatch using Bowtie1 option; library type, fr-second-strand; maximum intron length, 5000*; and transcript features and junction coordinates from the TAIR10 GTF (see Library Statistics section). The binary alignment and mapping files were then used for RPKM estimation and differential analysis using Cufflinks/CuffDiff v2.2.1 (9). The criteria for differentially accumulating nuclear-encoded protein-coding transcripts were as follows: RPKM > 5 in mutant or WT; fold change: > 2 in either direction (i.e. log2 > +1); and False Discovery Rate (FDR)-adjusted *P*<0.05 (Supplemental Dataset S1).

PARE and C-PARE libraries: Sequences were trimmed and processed using custom Perl scripts as described previously (11). The 20 nt reads were exactly matched to the Arabidopsis TAIR10 genome using Bowtie v0.12.8 (12), and short sequence reads or those matching to the genome > 20 times were removed. Remaining reads were matched to the TAIR10 representative protein-coding transcripts, with each mRNA concatenated with 100 nt of upstream sequence from the corresponding annotated TSS extracted from the genome (see Library Statistics section). Genome-matched trimmed reads were normalized to transcripts per 20 million reads (TP20M) for abundance comparisons.

C-PARE, WT and *xrn4-5* libraries were analyzed first to identify cap sites. For this analysis, the total PARE abundance was estimated by summing the abundance of PARE sequences on the positive strand of the transcript. MaxSeq was determined and Peak percent was calculated by dividing the MaxSeq abundance with the total PARE abundance of the transcript. MaxSeqs from C-PARE libraries were considered “cap sites” if they had an abundance > 5 TP20M, a peak percent > 10% and the same position between the two libraries. With these filters cap site positions for 14,010 transcripts (12,358 protein-coding transcripts) were identified and used in the analysis of PARE libraries. Since C-PARE is used only for positional information, we required the positions to be the same in both WT and *xrn4-5* and only these were considered to be the cap sites (Supplemental Figure S6B). This allowed greater confidence in identifying the cap sites due to the detectability in both backgrounds.

For the polyA+ and polyA- PARE libraries, the pipeline to identify decapped XRN4 substrates is outlined in Supplemental Figure S7. Briefly, MaxSeq abundance > 10 TP20M and peak percent > 5% were required for both *xrn4-5* and *xrn4-6* libraries. The non-redundant MaxSeqs with abundance fold change > 5 *xrn4*/WT and a position coincident with the cap site were identified as decapped XRN4 substrates.

For the 3’ fragment analysis, polyA+ MaxSeqs overaccumulating (>2 fold) at the same position (not incident with the cap site) in both *xrn4* mutants with an abundance > 20 TP20M and Peak percent > 5. A total of 568 sites (from 568 transcripts) were identified based on these criteria and were considered as 3’ fragments (includes seven miRNA targets). Only polyA+ PARE libraries were analyzed since 3’ fragments, in general, were hard to detect in polyA- PARE (see Figure 2B). The 3’ fragments (excluding the miRNA targets) were then compared to the list of CPuORF-containing, *upf1-* and *upf3*-elevated transcripts (Supplemental Dataset S2) to identify 3’ fragments associated with NMD-sensitive transcripts. Additionally, transcript PARE profiles were visually inspected using D-plots to exclude those with MaxSeqs that matched either the observed or annotated cap site. The NMD-sensitive 3’ fragments identified as XRN4 substrates are presented in Supplemental Dataset S2. For the analysis of miRNA targets (Figure 2, Supplemental Table S3), the list of 137 miRNA targets whose cleavage guided by miRNAs had been experimentally validated (5) plus *CCS1* (At1G12520) validated in (13) were examined and PARE abundance at the cleavage site was calculated as per legend to Figure 2B. All D-plots and PARE analyses are presented as abundance normalized to TP20M.

*Library Statistics*

*Summary statistics for RNA-seq:*

| Sample | RNA | *^1^* Total reads | Length | *^2^* Tophat aligned reads | *^3^* % Genome match | Library Name |
| --- | --- | --- | --- | --- | --- | --- |
| WT | PolyA+ | 27,258,425 | 51 | 27,151,285 | 96.9 | ATH599 |
| *xrn4-5* | PolyA+ | 24,343,237 | 51 | 24,247,079 | 96.9 | ATH600 |
| *xrn4-6* | PolyA+ | 30,919,076 | 51 | 29,944,440 | 97.2 | ATH601 |
| WT | PolyA- | 32,075,380 | 50 | 31,144,565 | 97.1 | ATH589 |
| *xrn4-5* | PolyA- | 33,898,451 | 50 | 32,951,149 | 97.2 | ATH590 |
| *xrn4-6* | PolyA- | 28,442,516 | 50 | 27,688,415 | 97.3 | ATH591 |
| WT | PolyA+ | 24,800,861 | 51 | 23,410,932 | 94.4 | ATH676 |
| *xrn4-5* | PolyA+ | 26,853,549 | 51 | 25,335,420 | 94.3 | ATH677 |
| *xrn4-6* | PolyA+ | 24,177,493 | 51 | 22,897,468 | 94.7 | ATH678 |
| WT | PolyA- | 36,097,563 | 50 | 34,356,556 | 95.2 | ATH566 |
| *xrn4-5* | PolyA- | 37,834,880 | 50 | 36,509,959 | 96.5 | ATH567 |
| *xrn4-6* | PolyA- | 27,075,111 | 50 | 26,370,007 | 97.4 | ATH568 |

***^1^*** Sequenced reads after adapter removal

***^2^*** Number of Tophat reads that matched the Arabidopsis TAIR10 genome

***^3^*** Percent of sequenced reads that matched the genome

*Summary statistics for PARE:*

| Library type | Sample | Trimmed 20mer distinct | Trimmed 20mer abundance | TAIR10 genome matched distinct | TAIR10 genome matched abundance | *^1^*TAIR10 cDNA matched distinct | TAIR10 cDNA matched abundance | Library name |
| --- | --- | --- | --- | --- | --- | --- | --- | --- |
| PARE | WT PolyA+ | 14,200,899 | 63,265,907 | 10,107,682 | 55,985,588 | 10,110,885 | 52,715,979 | ATH362 |
| PARE | *xrn4-5* PolyA+ | 12,230,666 | 80,268,147 | 8,194,731 | 71,680,067 | 8,244,187 | 59,514,983 | ATH363 |
| PARE | WT  PolyA+ | 10,523,341 | 37,315,147 | 8,049,527 | 33,068,634 | 8,151,714 | 31,644,947 | ATH237 |
| PARE | *xrn4-6* PolyA+ | 11,630,997 | 49,367,098 | 7,825,043 | 43,119,235 | 7,884,973 | 40,502,185 | ATH295 |
| PARE | WT PolyA- | 3,557,835 | 68,889,703 | 2,468,443 | 58,695,972 | 990,367 | 12,632,700 | ATH467 |
| PARE | *xrn4-5* PolyA- | 4,382,715 | 55,637,624 | 2,444,232 | 41,944,127 | 1,709,933 | 10,142,640 | ATH575 |
| PARE | WT PolyA- | 1,334,503 | 31,155,497 | 750,964 | 22,243,432 | 555,157 | 9,588,868 | ATH574 |
| PARE | *xrn4-6* PolyA- | 3,829,045 | 46,243,401 | 2,088,660 | 36,372,103 | 1,456,524 | 10,601,068 | ATH576 |
| C-PARE | WT PolyA+ | 3,415,421 | 52,094,603 | 1,218,296 | 43,950,280 | 1,125,545 | 43,474,897 | ATH292 |
| C-PARE | *xrn4-5* PolyA+ | 4,111,284 | 68,968,655 | 1,137,825 | 59,005,674 | 1,033,156 | 58,437,666 | ATH293 |

***^1^*** Reads matched to TAIR10 protein coding representative transcripts (cDNAs) that include 100 nt upstream sequence from each annotated transcription start site (TSS)

*Statistical analysis*

Data were analyzed with in-house Perl and R scripts (with Bioconductor packages). The heat maps and weighted Venn diagrams were generated using the *gplot* (*heatmap.2* function) and *VennDiagram* packages, respectively. Statistical significance between means were determined using analysis of variance (ANOVA) followed by multiple mean comparison tests (Figures 6C and 7B; Supplemental Figure S11) using Tukey’s HSD method (Origin 2019 program; [www.originlab.com](http://www.originlab.com)) or nonparametric Mann-Whitney U test (Figures 6D, 7D and Supplemental Figure S13).

Gene Ontology (GO) and overlap analysis: DAVID program (<http://david.abcc.ncifcrf.gov/>) was used for identifying enriched GO categories across polyA+ and polyA- decapped substrates of XRN4 from PARE compared against a background set of transcripts with an identified cap site. Categories with lowest P-values (*P<0.01,* polyA+; *P<0.01,* polyA-) obtained from Fisher Exact tests and fold enrichment > 2 are presented in Figure 5B. A complete list of all over-represented categories among decapped XRN4 substrates is presented in Supplemental Table S5. Categories enriched (*P<0.01*) among *xrn4*-elevated transcripts in polyA+ and polyA- RNA-seq are listed in Supplemental Table S5. Interactions between XRN4 substrates and different datasets were analyzed using the GeneSect tool (14) that measures the probability that the gene lists overlap is higher than would be expected by chance. Z-scores > 5 indicates overlap is significant. The datasets and the interaction results are summarized in Supplemental Table S4.

*Oligonucleotides*

| **Northern probes** | | |
| --- | --- | --- |
| **Primer** | **5’ to 3’ sequence** | **Description** |
| P5274 | CCCTAATCTGTTGTTTCTCGCTTCT | AT1G78080-F (RAP2.4/WIND1) |
| P5439 | CTTGTACGCCGCCTTATCAT | AT1G78080-R (RAP2.4/WIND1) |
| P5936 | AACAGTGTGAGCTACGTGAAAG | AT3G23030-F (IAA2) |
| P5937 | TGGATCTCTCGTCGGACAAA | AT3G23030-R (IAA2) |
| P5940 | GTCTCATAGTCACTCAGCTTCTC | AT5G20885-F |
| P5941 | CAGCTAGGTTCGTTCTTCGT | AT5G20885-R |
| P5942 | GAGGAGGGTACGATGAAGATTG | AT4G34760-F (SAUR LIKE) |
| P5943 | AACTTCTCTCACAGAACGAAGA | AT4G34760-R (SAUR LIKE) |
| P5944 | CAGGAACGGAGTCAAGTTTGGA | AT1G29920-F (CAB2) |
| P5946 | TACCATCCACCACAAACACAAACCTA | AT1G29920-R (CAB2) |
| P5578 | TACTTCCCGCTTACAGCAGTG | AT4G02970-F (AT7SL) |
| P5579 | GGACCAGCAGGCCATAGTG | AT4G02970-R (AT7SL) |
| P5570 | CGCAATCTTCGCTCTTCTCTTTGC | AT3G13920-F (eIF4A-1) |
| P5562 | CTCCCTTCACAGCAGATCG | AT3G13920-R (eIF4A-1) |
| P5922 | ATGGGCGTCGCTGTTCTAAATCC | AT4G32020-F |
| P5923 | AGGCGATGTCTAGGCGAAGGATC | AT4G32020-R |
| P6085 | TTCTCCTCCATATGATACATCCACC | AT5G47880-5’-F (eRF1-1) |
| P6086 | TGAGCGTTGTTCCTTCTTCTT | AT5G47880-5’-R (eRF1-1) |
| P6084 | CCAACAAGTCACAGGAAGGTTC | AT5G47880-3’-F (eRF1-1) |
| P6050 | ACCCAAATTCTCTCAGACAAGAG | AT5G47880-3’-R (eRF1-1) |
| P6092 | CTTGCACTATGGGAGGACTATG | AT5G19400-F (SMG7) |
| P6079 | ACCCAAGAGAGAGAGAGATAGC | AT5G19400-R (SMG7) |
| P5100 | GGAAATGGCTTTATATCTACTGACG | AT1G54490-F (XRN4) |
| P5186 | AGTTGATGACTGATCCCTCATCC | AT1G54490-R (XRN4) |
|  |  |  |
| PARE/ Anchor  RNA adapter | GUUCAGAGUUCUACAGUCCGAC |  |
|  |  |  |
| **5'RACE PCR** | | |
| **Primer** | **5' to 3' sequence** | **Description** |
| P6468 | CACCAATGAGCATCCAATCACCATC | AT1G04240_SHY2/IAA3 R |
| P5966 | CTCAGCTTCTCTGGATCATAAGG | AT3G23030_IAA2 R |

| **qPCR** | | | | |
| --- | --- | --- | --- | --- |
| **Primer** | **5' to 3' sequence** | **Primer Efficiency (%)** | **Amplicon** | **Description** |
| P5953 | CTACAGTCCGACATCAACCAGCTCACC | 92 | 153 nt | Splinted Ligation qRT-PCR AT3G23030-F (IAA2) |
| P5954 | GCTCGTTGACTTTCTCGTACGCC |  |  | Splinted Ligation qRT-PCR AT3G23030-R (IAA2) |
| P5952 | GTTCTTGGTGAGCTGGTTGATGTCGGACTGTAGAACTCT |  |  | Splint AT3G23030 (IAA2) |
| P6979 | CTACAGTCCGACATCTTCATCATCAGCAGCTTC | 91 | 227 nt | Splinted Ligation qRT-PCR AT1G04240-F (SHY2/IAA3) |
| P6524 | TCCTTGATGATGATTCAATCTCCTTCTCA |  |  | Splinted Ligation qRT-PCR AT1G04240-R (SHY2/IAA3) |
| P6978 | GAGAAGCTGCTGATGATGAAGATGTCGGACTGTAGAACTC |  |  | Splint AT1G04240 (SHY2/IAA3) |
| P5977 | GTTCTACAGTCCGACAGTTCATCGGTGAGGTT | 96 | 198 nt | Splinted Ligation qRT-PCR RAP2.4-F |
| P5978 | GAAGAAGCGAGAAACAACAGATTAGGG |  |  | Splinted Ligation qRT-PCR RAP2.4-R |
| P5976 | CTCTCAACCTCACCGATGAACTGTCGGACTGTAGAACTC |  |  | Splint RAP2.4 |
| P6263 | AGACGAGCTTAGGGTGTTGC | 101 | 134 nt | qRT-PCR AT1G76410-F (ATL8) |
| P6264 | CGCCACATTTATGACACCTG |  |  | qRT-PCR AT1G76410-R (ATL8) |
| P6012 | TCAGCGTTAAACTCGTCGTAGCAA | 95 | 88 nt | qRT-PCR AT5G49450-F (bZIP1) |
| P6013 | AACGCGGGTCTTAGATCGGAGAAG |  |  | qRT-PCR AT5G49450-R (bZIP1) |
| P5329 | CAGGTATCGCTGACCGTATGAG | 99 | 146 nt | qRT-PCR ACT2 F |
| P5330 | CATCTGCTGGAATGTGCTGAGG |  |  | qRT-PCR ACT2 R |
| P5289 | AGTCATCAGCTCGCGTTGAC | 98 | 67 nt | qRT-PCR 18S F |
| P5290 | TCAATCGGTAGGAGCGACG |  |  | qRT-PCR 18S R |
| P5944 | CAGGAACGGAGTCAAGTTTGGA | 93 | 106 nt | qRT-PCR CAB2 F |
| P5946 | TACCATCCACCACAAACACAAACCTA |  |  | qRT-PCR CAB2 R |
| P5709 | CTGTAATCCCTTAGATCTTC | 97 | 183 nt | qRT-PCR GRP7 F |
| P6276 | TCCCCTTGATCTTCCAGTC |  |  | qRT-PCR GRP7 R |
| P5561 | ATTTCGTGACCCGTGATGAT | 98 | 143 nt | qRT-PCR eIF4-A F |
| P5562 | CTCCCTTCACAGCAGATCG |  |  | qRT-PCR eIF4-A R |
| P5103 | TTCTACAGTCCGACCTCTTTCGGATATTCCACCAG | 94 | 133 nt | Splinted Ligation qRT-PCR eIF4-A F |
| P5104 | AGTTGGTAAGCGAGAGTAAAGG |  |  | Splinted Ligation qRT-PCR eIF4-A R |
| P5255 | TGGTGGAATATCCGAAAGAGGTCGGACTGTAGAA |  |  | Splint eIF4-A |
| P5095 | CTGCGACTCAGGGAATCTTCTAA | 99 | 62 nt | qRT-PCR AT5G25760-F (UBC) |
| P5096 | TTGTGCCATTGAATTGAACCC |  |  | qRT-PCR AT5G25760-R (UBC) |

**Supporting References**

1. Schmidt,S.A., Foley,P.L., Jeong,D.-H., Rymarquis,L.A., Doyle,F., Tenenbaum,S.A., Belasco,J.G. and Green,P.J. (2015) Identification of SMG6 cleavage sites and a preferred RNA cleavage motif by global analysis of endogenous NMD targets in human cells. *Nucleic Acids Res.*, **43**, 309–323.

2. Sorenson,R.S., Deshotel,M.J., Johnson,K., Adler,F.R. and Sieburth,L.E. (2018) Arabidopsis mRNA decay landscape arises from specialized RNA decay substrates, decapping-mediated feedback, and redundancy. *Proc. Natl. Acad. Sci. U. S. A.*, **115**, E1485–E1494.

3. German,M.A., Luo,S., Schroth,G., Meyers,B.C. and Green,P.J. (2009) Construction of Parallel Analysis of RNA Ends (PARE) libraries for the study of cleaved miRNA targets and the RNA degradome. *Nat. Protoc.*, **4**, 356–362.

4. German,M.A., Pillay,M., Jeong,D.H., Hetawal,A., Luo,S., Janardhanan,P., Kannan,V., Rymarquis,L.A., Nobuta,K., German,R., *et al.* (2008) Global identification of microRNA-target RNA pairs by parallel analysis of RNA ends. *Nat Biotechnol*, **26**, 941–946.

5. Jeong,D.-H., Schmidt,S., Rymarquis,L., Park,S., Ganssmann,M., German,M., Accerbi,M., Zhai,J., Fahlgren,N., Fox,S., *et al.* (2013) Parallel analysis of RNA ends enhances global investigation of microRNAs and target RNAs of Brachypodium distachyon. *Genome Biol.*, **14**, R145.

6. Jeong,D.H. and Green,P.J. (2012) Methods for validation of miRNA sequence variants and the cleavage of their targets. *Methods*, **58**, 135–143.

7. Llave,C., Kasschau,K.D., Rector,M.A. and Carrington,J.C. (2002) Endogenous and silencing-associated small RNAs in plants. *Plant Cell*, **14**, 1605–1619.

8. Harigaya,Y. and Parker,R. (2012) Global analysis of mRNA decay intermediates in Saccharomyces cerevisiae. *Proc. Natl. Acad. Sci.*, **109**, 11764–11769.

9. Trapnell,C., Roberts,A., Goff,L., Pertea,G., Kim,D., Kelley,D.R., Pimentel,H., Salzberg,S.L., Rinn,J.L. and Pachter,L. (2012) Differential gene and transcript expression analysis of RNA-seq experiments with TopHat and Cufflinks. *Nat. Protoc.*, **7**, 562–578.

10. Trapnell,C., Pachter,L. and Salzberg,S.L. (2009) TopHat: discovering splice junctions with RNA-Seq. *Bioinformatics*, **25**, 1105–1111.

11. Rymarquis,L.A., Souret,F.F. and Green,P.J. (2011) Evidence that XRN4, an Arabidopsis homolog of exoribonuclease XRN1, preferentially impacts transcripts with certain sequences or in particular functional categories. *RNA*, **17**, 501–511.

12. Langmead,B., Trapnell,C., Pop,M. and Salzberg,S. (2009) Ultrafast and memory-efficient alignment of short DNA sequences to the human genome. *Genome Biol.*, **10**, R25.

13. Beauclair,L., Yu,A. and Bouché,N. (2010) microRNA-directed cleavage and translational repression of the copper chaperone for superoxide dismutase mRNA in Arabidopsis. *Plant J.*, **62**, 454–462.

14. Katari,M.S., Nowicki,S.D., Aceituno,F.F., Nero,D., Kelfer,J., Thompson,L.P., Cabello,J.M., Davidson,R.S., Goldberg,A.P., Shasha,D.E., *et al.* (2010) VirtualPlant: A Software Platform to Support Systems Biology Research. *Plant Physiol.*, **152**, 500–515.
