# Supplementary material for: RNA degradomes reveal substrates and importance for dark and nitrogen stress responses of Arabidopsis XRN4
Source: Nucleic Acids Res. 2019 Aug 20;47(17):9216–30. doi: 10.1093/nar/gkz712 (PMC6755094; doi:10.1093/nar/gkz712)
Supplement: gkz712_Supplemental_Files [file gkz712_supplemental_files.zip › Supplementary Datasets.pdf]

Supplementary Dataset 1. Transcripts differentially accumulating in *xrn4* mutants as compared to WT in polyA+ and polyA- RNA-seq

Supplementary Dataset 2. Endogenous substrates of Arabidopsis XRN4

Supplementary Tables, Figures and Experimental Procedures
